# Molecular detection of zoonotic pathogens in fleas collected from dogs and cats in Portugal

**DOI:** 10.1186/s13071-026-07456-4

**Published:** 2026-05-30

**Authors:** André Pereira, Adrian Cruz, João Cruz, David Garcia-Dios, Teresa Novo, Carla Maia

**Affiliations:** 1 Research in Veterinary Medicine, I-MVET, Faculty of Veterinary Medicine, Lusófona University-Lisbon University Centre, Campo Grande nº 376, 1749-024 Lisbon, Portugal; 2 Animal and Veterinary Research Center, CECAV, Faculty of Veterinary Medicine, Lusófona University-Lisbon University Centre, Campo Grande nº 376, 1749-024 Lisbon, Portugal; 3 Superior School of Health, Protection and Animal Welfare, ESPA, Polytechnic Institute of Lusophony, IPLUSO, Rua do Telhal aos Olivais nº 8, 1950-396 Lisbon, Portugal; 4 Global Health and Tropical Medicine, GHTM, LA-REAL, Instituto de Higiene e Medicina Tropical, IHMT, Universidade NOVA de Lisboa, Rua da Junqueira nº100, 1349-008 Lisbon, Portugal; 5 IHMT, Universidade NOVA de Lisboa, Rua da Junqueira nº100, 1349-008 Lisbon, Portugal; 6 Investigación en Sanidad Animal: Galicia (INVESAGA) Faculty of Veterinary Sciences, Universidade de Santiago de Compostela, Carvalho Calero, 27002 Lugo, Spain

**Keywords:** *Bartonella*, Cats, *Dipylidium caninum*, Dogs, Flea infestations, *Rickettsia*, One health, Portugal

## Abstract

**Background:**

Fleas are common ectoparasites of companion animals and key vectors of pathogens of veterinary and zoonotic relevance. Although flea infestations are highly prevalent in dogs and cats in Portugal, nationwide data on flea-borne pathogens are limited. This study aimed to estimate the prevalence and characterize the molecular diversity of *Bartonella* spp., *Rickettsia* spp., and *Dipylidium caninum* in fleas collected from dogs and cats in mainland Portugal, and to identify associated epidemiological factors.

**Methods:**

Fleas collected from dogs and cats across mainland Portugal were screened using conventional polymerase chain reaction (PCR) assays targeting the 16S–23S ribosomal RNA (rRNA) intergenic spacer region of *Bartonella* spp., the *ompB* gene of *Rickettsia* spp., and the 28S rRNA gene region of *D. caninum*. Positive amplicons were sequenced and analyzed phylogenetically. Associations between pathogen detection and epidemiological variables, including host species, host lifestyle, flea species, and geographic region, were evaluated using multivariable logistic regression models.

**Results:**

A total of 1261 fleas were analyzed, with 24.7% positive for at least one pathogen. *Bartonella* spp. DNA was detected in 8.3% of fleas and was significantly more frequent in fleas from cats, particularly stray/sheltered animals, with regional differences also observed. Identified species included *B. clarridgeiae*, *B. henselae*, and *B. rochalimae*. *Rickettsia* spp. DNA was detected in 16.2% of fleas, predominantly in *Ctenocephalides felis*, with flea species, host species, and geographic region identified as independent predictors of positivity. Most sequences corresponded to *R. felis*, while *R. asembonensis* and *Candidatus* R. senegalensis, the latter reported for the first time in Europe, were also identified. *Dipylidium caninum* DNA was detected in 1.8% of fleas, all *C. felis*, with both canine- and feline-associated genotypes circulating in fleas from dogs and cats. Co-detection of pathogen DNA occurred in 2.1% of fleas, mostly *Bartonella* spp./*Rickettsia* spp.

**Conclusions:**

Fleas infesting companion animals in Portugal frequently harbor pathogens of veterinary and zoonotic relevance, with *C. felis* playing a central role in pathogen circulation at the human–animal interface. These findings reinforce the need for sustained surveillance and effective ectoparasite control within a One Health framework to reduce pathogen circulation and mitigate risks to both animal and public health.

**Graphical abstract:**

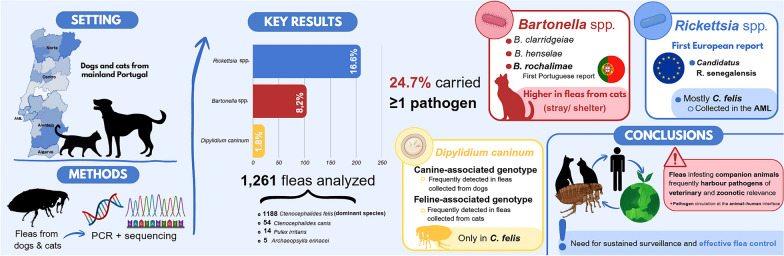

**Supplementary Information:**

The online version contains supplementary material available at 10.1186/s13071-026-07456-4.

## Background

Fleas (Insecta: Siphonaptera) are among the most common hematophagous ectoparasites of companion animals and are of major veterinary and public health importance due to their role as vectors and intermediate hosts of pathogens of zoonotic concern [1–3]. *Ctenocephalides felis*, the cat flea, is recognized as the predominant species infesting dogs and cats worldwide [4, 5]. Molecular studies have further revealed marked intraspecific genetic diversity within *C. felis*, with distinct clades showing differential associations with specific pathogens and endosymbionts, suggesting that vector genetic background may influence pathogen infection patterns [6]. In southern European countries, including Portugal, climatic conditions characterized by mild winters and mild-hot summers favor year-round flea survival and activity, resulting in sustained infestation pressure in domestic animal populations [1, 7, 8]. Through their close association with pets that share human environments, fleas facilitate the circulation of vector-borne pathogens at the animal–human interface [2, 3, 9, 10].

Among flea-borne pathogens affecting dogs and cats, the bacteria *Bartonella* spp. and *Rickettsia* spp., and the cestode *Dipylidium caninum* are of particular epidemiological relevance [4, 11–13]. Fleas are recognized vectors of several zoonotic *Bartonella* spp., including *B. henselae*, *B. clarridgeiae*, and *B. rochalimae*, which are frequently detected in fleas collected from dogs and cats and are associated with cat-scratch disease and other bartonelloses in humans, with clinical manifestations ranging from self-limited febrile illness to severe complications, including blood culture-negative endocarditis, bacillary angiomatosis, and neuroretinitis, particularly in immunocompromised patients [14–19]. Domestic cats act as the primary reservoir hosts, while flea infestation is central to pathogen transmission within feline populations and to spillover risk at the human–animal interface [20, 21].

*Rickettsia felis*, the etiological agent of flea-borne spotted fever, shows a strong bio-ecological association with *C. felis* infesting companion animals and is now recognized as an emerging cause of febrile illness in humans in many regions, including Europe [22, 23]. In addition to acting as vectors, *C. felis* fleas maintain *R. felis* through transstadial and transovarial transmission, supporting their role as invertebrate reservoir hosts for this pathogen [5, 24]. Experimental evidence further demonstrates that dogs can develop prolonged subclinical rickettsemia following *R. felis* infection and efficiently infect naïve fleas feeding on them, supporting their role as competent vertebrate reservoir hosts [19, 25]. Furthermore, fleas can harbor other potentially zoonotic *Rickettsia* spp., such as *Rickettsia asembonensis*, which have been detected in fleas from companion animals and wildlife across diverse ecological contexts [26–29].

*Dipylidium caninum* relies on fleas and, less frequently, lice as intermediate hosts, with infection of dogs, cats, and occasionally humans occurring through ingestion of infected ectoparasites [11, 30]. In dogs and cats, infection is often subclinical, but may cause perianal pruritus, gastrointestinal signs, and weight loss, particularly in young or heavily infected animals [11]. In humans, especially infants and young children, infection is typically benign and characterized by the passage of motile proglottids, occasionally accompanied by abdominal discomfort and anal pruritus [11]. Although molecular surveys generally report lower frequencies of *D. caninum* in fleas compared with bacterial pathogens [3, 31, 32], its consistent detection in fleas collected from dogs and cats confirms an ongoing role of these ectoparasites in maintaining the parasite’s life cycle and sustaining transmission [11]. Molecular studies further indicate the existence of genetically distinct canine- and feline-associated *D. caninum* genotypes, suggesting vertebrate host-linked lineages and possible host-specific adaptations [30, 33].

Overall, several studies demonstrate that a substantial proportion of *C. felis* fleas collected from dogs and cats carry *Bartonella* spp. and/or *Rickettsia* spp., with infection patterns influenced by host species, management practices, and geographical context [3, 10, 34–36]. Such findings emphasize the importance of region-specific data to support surveillance and control strategies for flea-borne pathogens within a One Health framework [2].

Despite growing global recognition of flea-borne infections, comprehensive nationwide data on the occurrence and molecular diversity of *Bartonella* spp., *Rickettsia* spp., and *D. caninum* in fleas from dogs and cats remain scarce in Portugal [37], where *C. felis* infestations are highly prevalent [1]. Consequently, the distribution of these pathogens and their associations with host-related, geographic, and flea species-related factors remain insufficiently characterized, limiting evidence-based risk assessment and control planning.

Therefore, this study aimed to estimate the molecular prevalence and explore genetic diversity of *Bartonella* spp., *Rickettsia* spp. and *D. caninum* in fleas collected from dogs and cats in Portugal, and to evaluate the association of flea species, host species, host lifestyle, and geographic region with pathogen occurrence in fleas.

## Methods

### Study design and flea sampling

The present study used flea specimens obtained within the framework of a nationwide epidemiological survey of flea infestations in dogs and cats from Portugal, as described by Pereira et al. [1]. Briefly, fleas were collected from client-owned, shelter and stray dogs and cats across the five Nomenclature of Units for Territorial Statistics II (NUTS II) region (Norte, Centro, Área Metropolitana de Lisboa [AML], Alentejo and Algarve, Portugal) of mainland Portugal.

Fleas were primarily identified to species level on the basis of morphological examination using taxonomic keys, with molecular methods applied to confirm species identification in a subset of specimens, as detailed in Pereira et al. [1].

To minimize host-related clustering and overrepresentation of heavily infested animals, a subsampling strategy was applied. For hosts from which more than three fleas were collected, three fleas plus an additional 20% of the total number of fleas collected from that host were selected for molecular screening. When three or fewer fleas were collected, all specimens were analyzed.

### DNA extraction

Genomic DNA was extracted from individual fleas using the NZY Tissue gDNA Isolation kit (NZYTech, Lisbon, Portugal), following the methodology described in Pereira et al. [1] with modifications. Briefly, each specimen was transferred to a 1.5 ml sterile tube containing 180 µl of NT1 buffer (NZYTech) and manually macerated using a sterile disposable pestle, after which 25 µl of proteinase K (20 mg/ml; NZYTech) was added. Samples were incubated at 56 °C for 3 h with periodic agitation; after 90 min, an additional 16 µl of proteinase K (50 mg/ml; GRiSP, Porto, Portugal) was added. DNA extraction was completed according to the manufacturer’s instructions. Extracted DNA was stored at −20 °C until further analysis.

### PCR-based detection of flea-borne pathogens

Flea specimens were screened for the presence of *Bartonella* spp., *Rickettsia* spp., and *D. caninum* DNA using pathogen-specific conventional polymerase chain reaction (PCR) protocols (Table 1) [38–40]. An initial screening was performed using randomly assembled DNA pools of five fleas, with 1 µl of DNA per specimen contributing to a total of 5 µl of pooled DNA per reaction; the same PCR conditions were applied for both pooled and individual screening. Fleas from pools yielding detectable PCR amplification were subsequently analyzed individually, using 5 µl of DNA per reaction. PCR products were separated by electrophoresis on 1.5% agarose gels stained with GreenSafe Premium (NZYTech) and visualized under ultraviolet illumination; fragment sizes were assessed by comparison with a 100 bp DNA ladder (NZYTech). Positive controls consisting of genomic DNA from *R. conorii*, *D. caninum*, and *B. henselae*, and negative controls containing nuclease-free, ultrapure water instead of template DNA, were included in each PCR run.

**Table 1:** PCR protocols performed for the molecular detection and characterization of flea-borne pathogens

Pathogen (molecular target)	Primer sequence (5′–3′)	Amplicon size (bp)	Reaction setup	Thermocycling conditions	References
*Bartonella* spp.	Fw (325 s): CTTCAGATGATGATCCCAAGCCTTCTGGCG	453–800	25 µl reaction: 5 µl of DNA; 0.4 µM of each primer; 12.5 µl of NZYTaq 2 × Green Master Mix	95 °C—5 min; 55 cycles [95 °C—30 s; 66 ºC—30 s; 72 °C—45 s]; 72 °C—5 min	[40]
(16S–23S rRNA ITS)	Rev (1100as): GAACCGACGACCCCCTGCTTGCAAAGCA				
*Rickettsia* spp.	Fw (120–2788): AAACAATAATCAAGGTACTGT	~ 800		95 °C—3 min; 40 cycles [95 °C—30 s; 50 °C—30 s; 68 °C—90 s]; 68 °C—7 min	[38]
(*ompB*)	Rev (120–3599): TACTTCCGGTTACAGCAAAGT				
*Dipylidium caninum*	Fw (DC28S-1F): GCATGCAAGTCAAAGGGTCCTACG	653		94 °C—5 min; 30 cycles [94 °C—30 s; 52 °C 30 s; 72 °C – 40 s]; 72 °C—10 min	[39]
(28S rRNA)	Rev (DC28S-1R): CACATTCAACGCCCGACTCCTGTAG				

*bp* base pairs, *Fw* forward, *ITS* internal transcribed spacer, *ompB*, outer membrane protein B, *rRNA* ribosomal RNA, *Rev* reverse

### Sequencing and bioinformatic analyses

Positive PCR products were purified using the Speedy NZY ExoSAP Mix kit (NZYTech) and sequenced by the Sanger method at STAB VIDA (Caparica, Portugal), using the respective forward primers employed for DNA amplification. Obtained sequences were compared with sequences available in the GenBank database using the Basic local alignment search tool (BLASTn) tool (http://blast.ncbi.nlm.nih.gov/Blast.cgi).

Multiple sequence alignments were generated using the iterative G-INS-i refinement method implemented in MAFFT v7 [41]. Alignments were subsequently processed with Gblocks [42] via SeaView v5.0.5 using default parameters and, when applicable, manually curated using AliView v1.28 to ensure preservation of correct reading frames. Sequences with insufficient length or poor quality, resulting in excessive missing or ambiguous bases in the final alignment, were excluded from phylogenetic analyses.

Phylogenetic analyses were performed using the maximum likelihood method implemented in IQ-TREE v2.4.0 [43]. The best-fit nucleotide substitution model was selected according to the Bayesian Information Criterion, and branch support was assessed by bootstrap resampling with 1000 replicates; bootstrap values ≥ 75 were considered indicative of robust topological support. Resulting phylogenetic trees were visualized and edited using FigTree v.1.4.4.

Nucleotide sequences obtained in this study were deposited in the DDBJ/ENA/GenBank databases under the accession numbers LC914712–LC914732 (*D. caninum*), LC914763–LC914966 (*Bartonella* spp.), and LC915091–LC915184 (*Rickettsia* spp.). All sequences included in the phylogenetic analyses and associated metadata are listed in Additional file 1: Tables S1-3.

### Statistical analysis

Statistical analyses were performed using IBM^®^ SPSS^®^ Statistics v25.0. Pathogen prevalence was calculated as the proportion of PCR-positive fleas among the total number of fleas tested for each agent, and 95% confidence intervals (CI) were determined using Wilson’s method.

Associations between PCR positivity for *Bartonella* spp., *Rickettsia* spp. and *D. caninum* and explanatory variables (i.e., flea species, host, host lifestyle, geographic region, and parish type), were initially evaluated using Pearson’s chi-square (*χ*^2^) test or the alternative Fisher’s exact test for 2 × 2 tables or Fisher–Freeman–Halton test for larger contingency tables.

Adjusted standardized residuals (ASR) were calculated, with values exceeding ± 1.96 considered indicative of statistically significant contributions at *α* = 0.05.

Variables with *P* ≤ 0.20 in univariable analyses were considered for inclusion in multivariable logistic regression models to identify independent predictors of pathogen detection in fleas. A backward stepwise elimination procedure was applied, with variables retained in the final model at *P* ≤ 0.05. Results are presented as adjusted odds ratios (aOR) with 95% CI. Overall model significance was assessed using the likelihood ratio (*G*^*2*^) test, while model calibration and discrimination were evaluated using the Hosmer–Lemeshow goodness-of-fit test and the area under the receiver operating characteristic curve (AUC), respectively.

Choropleth maps were generated to depict the spatial distribution of *Rickettsia* spp., *Bartonella* spp., and *D. caninum* prevalence in fleas across mainland Portugal, stratified by NUTS II regions. Mapping was performed in QGIS v3.44.4 (Solothurn) using administrative boundary layers obtained from the Geographic Information System of the European Commission (GISCO; https://ec.europa.eu/eurostat/web/gisco/geodata/administrative-units/countries).

## Results

**Fig. 1:**
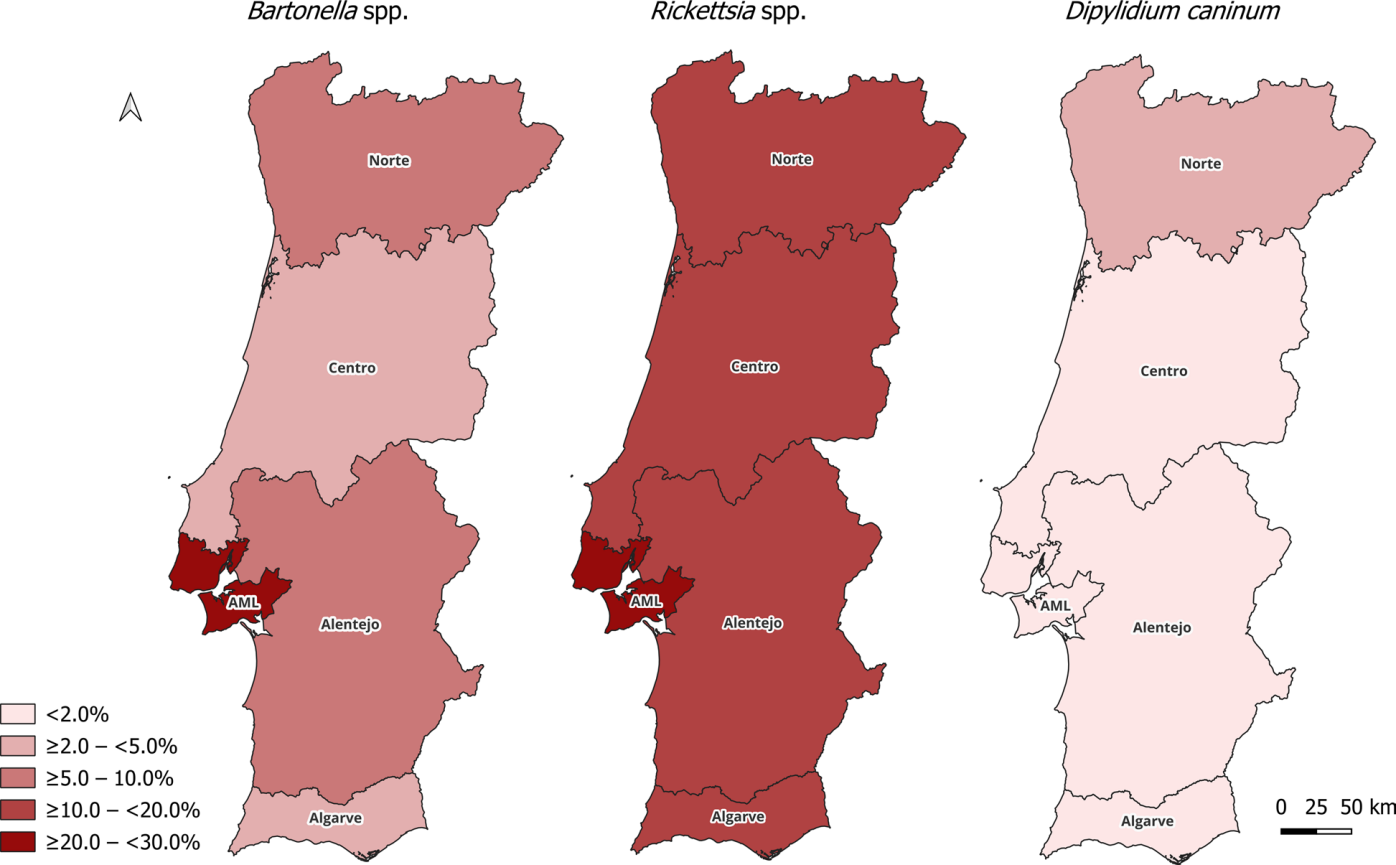
Geographical distribution of the molecular prevalence of *Bartonella* spp., *Rickettsia* spp. and *Dipylidium caninum* in fleas collected from dogs and cats across mainland Portugal, stratified by NUTS II regions. Regions are color-coded according to prevalence categories, as indicated in the legend. AML, Área Metropolitana de Lisboa

A total of 1261 fleas collected from dogs (*n* = 656, 52.0%) and cats (*n* = 605, 48.0%) in mainland Portugal, comprising *C. felis* (*n* = 1,188, 94.2%), *Ctenocephalides canis* (*n* = 54, 4.3%), *Pulex irritans* (*n* = 14, 1.1%), and *Archaeopsylla erinacei* (*n* = 5, 0.4%), were screened by PCR for *Bartonella* spp., *Rickettsia* spp. and *D. caninum*. Overall, 311 fleas (24.7%) tested positive for at least one pathogen, with the geographical distribution of molecular prevalence shown in Fig. 1.

### *Bartonella* spp.

*Bartonella* spp. DNA was detected in 105/1261 fleas (8.3%, 95% CI 6.9–10.0) and was significantly more frequent in fleas collected from cats (14.9%, 95% CI 12.4–18.1; *χ*^2^ = 65.35, *df* = 1, *P* < 0.0001) than from dogs (2.3%, 95% CI 1.7–4.3) (Table 2).

**Table 2:** Prevalence of *Bartonella* spp., *Rickettsia* spp. and *Dipylidium caninum* in fleas collected from dogs and cats in mainland Portugal according to host and flea characteristics

Variable/categories	Tested		*Bartonella* spp.				*Rickettsia* spp.				*Dipylidium caninum*			
	*n*	%	*n*	%	IC95%	*P*-value	*n*	%	IC95%	*P*-value	*n*	%	IC95%	*P*-value
Flea species	1261					0.193				0.032				0.733
*Archaeopsylla erinacei*	5	0.4	0	0.0	0.0–43.5		0	0.0	0.0–43.5		0	0.0	0.0–43.5	
*Ctenocephalides canis*	54	4.3	1	1.9	0.3–9.8		2	3.7^b^	1.0–12.5		0	0.0	0.0–6.6	
*Ctenocephalides felis*	1188	94.2	102	8.6	7.1–10.3		200	16.8^a^	14.8–19.1		23	1.9	1.3–2.9	
*Pulex irritans*	14	1.1	2	14.3	4.0–39.9		2	14.3	4.0–39.9		0	0.0	0.0–21.5	
Host	1261					< 0.001				0.146				0.834
Dog	656	52.0	15	2.3^b^	1.7–4.3		116	17.7	15.0–20.8		11	1.7	0.9–3.0	
Cat	605	48.0	90	14.9^a^	12.4–18.1		88	14.5	12.0–17.6		12	2.0	1.1–3.4	
Host lifestyle	1236					< 0.001				0.072				0.153
Sheltered	369	29.9	32	8.7	6.2–12.0		68	18.4	14.8–22.7		13	3.5	2.1–5.9	
Stray	122	9.9	28	23.0^a^	16.4–31.2		14	11.5	7.0–18.3		1	0.8	0.1–4.5	
Domestic indoor	113	9.1	0	0.0^b^	0.0–3.3		10	8.8	4.9–15-5		1	0.9	0.2–4.8	
Domestic indoor-outdoor	457	37.0	32	7.0	5.0–9.7		80	17.5	14.3–21.3		5	1.1	0.5–2.5	
Domestic outdoor	175	14.2	11	6.3	3.5–10.9		31	17.7	12.8–24.1		2	1.1	0.3–4.1	
Region-NUTS II	1250					< 0.001				0.032				0.100
Norte	471	37.7	47	10.0	7.6–13.0		62	13.2^b^	10.4–16.5		13	2.8	1.6–4.7	
Centro	248	19.8	10	4.0^b^	2.2–7.3		44	17.7	13.5–23.0		2	0.8	0.2–2.9	
AML	96	7.7	23	24.0^a^	16.5–33.4		25	26.0^a^	18.3–35.6		1	1.0	0.2–5.7	
Alentejo	188	15.0	18	9.6	6.1–14.6		32	17.0	12.3–23.0		3	1.6	0.5–4.6	
Algarve	247	19.8	7	2.8^b^	1.4–5.7		40	16.2	12.1–21.3		4	1.6	0.6–4.1	
Parish type	1167					0.001				0.337				0.196
Non-rural	565	48.4	64	11.3^a^	9.0–14.2		85	15.0	12.3–18.2		14	2.5	1.5–4.1	
Rural	602	51.6	36	6.0^b^	4.4–8.2		103	17.1	14.3–20.3		8	1.3	0.7–2.6	
Total	1261		105	8.3	6.9–10.0		204	16.2	14.3–18.3		23	1.8	1.2–2.7	

^a^Adjusted standardized residual ≥  +1.96, significantly lower than expected

^b^Adjusted standardized residual ≤  −1.96, significantly lower than expected

*CI* confidence interval, *NUTS* nomenclature of units for territorial statistics

According to host lifestyle, the highest prevalence was observed in fleas from stray animals (23.0%, 95% CI 16.4–31.2; *χ*^2^ = 46.47, *df* = 4, *P* < 0.0001), followed by sheltered animals (8.7%, 95% CI 6.2–12.0). *Bartonella* spp. DNA was not detected in fleas from strictly indoor domestic animals.

In the multivariable logistic regression model, *Bartonella* spp. DNA detection was independently associated with host species, host lifestyle, and geographic region (*G*^2^ = 112.75, *df* = 6, *P* < 0.0001) (Additional file 2: Table S1). Fleas collected from cats had significantly higher odds of infection than those from dogs (aOR = 6.95, 95% CI 3.87–12.49, Wald *χ*^2^ = 42.09, *df* = 1, *P* < 0.0001). Fleas from sheltered or stray animals were more likely to be positive compared with those from domestic animals (aOR = 1.75, 95% CI 1.10–2.78; Wald* χ*^2^ = 5.55, *df* = 1, *P* = 0.019).

Significantly lower odds of *Bartonella* spp. positivity were observed in the Norte (aOR = 0.45, 95% CI 0.25–0.81; Wald *χ*^2^ = 7.05, *df* = 1, *P* = 0.008), Centro (aOR = 0.23, 95% CI 0.10–0.53; Wald* χ*^2^ = 11.98, *df* = 1, *P* = 0.001), Alentejo (aOR = 0.36, 95% CI 0.18–0.73; Wald* χ*^2^ = 7.96, *df* = 1, *P* = 0.005), and Algarve (aOR = 0.10, 95% CI 0.04–0.25; Wald* χ*^2^ = 24.2, *df* = 1, *P* < 0.0001) regions compared with AML.

**Fig. 2:**
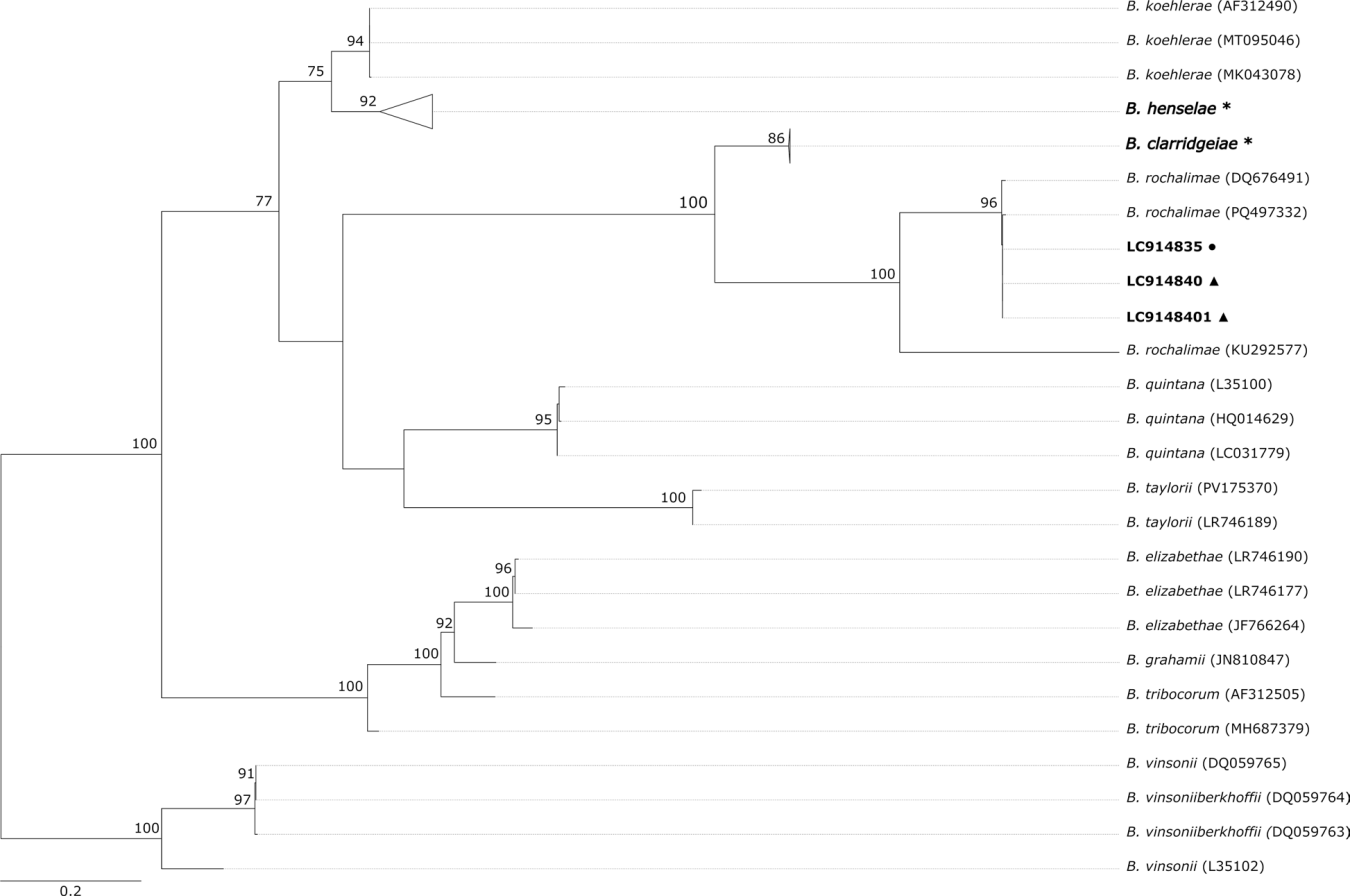
Maximum likelihood phylogenetic tree inferred from 16S to 23S rRNA intergenic spacer region sequences of *Bartonella* spp. detected in fleas collected from dogs and cats in Portugal. Tree reconstruction was performed in IQ-TREE using the best-fitting nucleotide substitution model selected according to the Bayesian Information Criterion. Node support was assessed using 1000 bootstrap replicates, and values ≥ 75% are shown at the corresponding nodes. Reference sequences are labeled with species designation and GenBank accession numbers. Sequences obtained in this study are indicated in bold by their respective GenBank accession numbers; circle symbols denote sequences obtained from *Ctenocephalides felis*, whereas triangle symbols indicate sequences obtained from *Pulex irritans*. Owing to the large number of sequences included, the *B. henselae* and *B. clarridgeiae* clusters were collapsed and are indicated in bold by an asterisk. The complete tree with the *B. henselae* and *B. clarridgeiae* clusters expanded is provided in Additional file 3: Fig. S1. Associated sequence metadata are provided in Additional file 1: Tables S1. Branch lengths are scaled to the number of substitutions per site

Phylogenetic analysis enabled species-level characterization of most *Bartonella*-positive sequences, revealing the presence of *B. clarridgeiae* (*n* = 62), *B. henselae* (*n* = 39) and *B. rochalimae* (*n* = 3) (Table 3). All analyzed sequences segregated into well-supported monophyletic clades together with their corresponding reference sequences (Fig. 2; Additional file 3: Fig. S1). The remaining sequence, confirmed as *Bartonella* spp. by BLASTn analysis, was too short (256 bp) for reliable phylogenetic placement and unambiguous species-level identification.

**Table 3:** Prevalence of *Bartonella* spp., *Rickettsia* spp., *Dipylidium caninum*, and co-detections in fleas collected from dogs and cats in mainland Portugal, stratified by flea species

Pathogen^a^	*Ctenocephalides felis*, *n* (%)	*Ctenocephalides canis*, *n* (%)	*Pulex irritans*, *n* (%)	Total, *n* (%)
*Bartonella* spp.^b^	**102 (8.6)**	**1 (1.9)**	**2 (14.3)**	**105 (8.3)**
*B. clarridgeiae*	62 (60.8)	0 (0.0)	0 (0.0)	62 (59.0)
*B. henselae*	38 (37.3)	1 (100.0)	0 (0.0)	39 (37.1)
*B. rochalimae*	1 (1.0)	0 (0.0)	2 (100.0)	3 (2.9)
*Bartonella* sp.^c^	1 (1.0)	0 (0.0)	0 (0.0)	1 (1.0)
*Rickettsia* spp.^b^	**200 (16.8)**	**2 (3.7)**	**2 (14.3)**	**204 (16.2)**
*R. felis*	189 (94.5)	1 (50.0)	2 (100.0)	192 (94.1)
*R. asembonensis*	1 (0.5)	0 (0.0)	0 (0.0)	1 (0.5)
*Candidatus* R. senegalensis	1 (0.5)	0 (0.0)	0 (0.0)	1 (0.5)
*Rickettsia* sp.^c^	9 (4.5)	1 (50.0)	0 (0.0)	10 (4.9)
*Dipylidium caninum*^b^	**23 (1.9)**	**0 (0.0)**	**0 (0.0)**	**23 (1.8)**
Canine genotype	10 (43.5)	0 (0.0)	0 (0.0)	10 (43.5)
Feline genotype	11 (47.8)	0 (0.0)	0 (0.0)	11 (47.8)
Ungenotyped	2 (8.7)	0 (0.0)	0 (0.0)	2 (8.7)
Co-detections^b^	**26 (2.2)**	**0 (0.0)**	**1 (7.1)**	**27 (2.1)**
*B. clarridgeiae* + *R. felis*	12 (46.2)	0 (0.0)	0 (0.0)	12 (1.0)
*B. henselae* + *R. felis*	10 (38.5)	0 (0.0)	0 (0.0)	10 (0.8)
*B. rochalimae* + *R felis*	0 (0.0)	0 (0.0)	1 (100.0)	1 (0.1)
*B. clarridgeiae* + *D. caninum*	1 (3.8)	0 (0.0)	0 (0.0)	1 (0.1)
*B. henselae* + *D. caninum*	1 (3.8)	0 (0.0)	0 (0.0)	1 (0.1)
*R. felis* + *D. caninum*	1 (3.8)	0 (0.0)	0 (0.0)	1 (0.1)
*Candidatus* R. senegalensis + *D. caninum*	1 (3.8)	0 (0.0)	0 (0.0)	1 (0.1)

^a^No pathogens were detected in *Archaeopsylla erinacei* (*n* = 5)

^b^Bold values indicate overall group-level prevalence. Percentages for the overall prevalence of *Bartonella* spp., *Rickettsia* spp., and *D. caninum*, as well as for co-detections, were calculated using the total number of fleas tested for each flea species (*C. felis*,* n* = 1,188; *C. canis*, *n* = 54; *P. irritans*, *n* = 14). In the Total column, percentages were calculated using the total number of fleas analyzed (*N* = 1,261). For individual pathogen species, *D. caninum* genotypes and specific co-detection combinations, percentages were calculated relative to the number of fleas positive for the corresponding pathogen group or co-detection category. Fleas positive for more than one pathogen are included in the prevalence estimate of each genus involved, and additionally listed once in the co-detection section

^c^Species or genotype-level identification could not be achieved owing to insufficient sequence resolution

### *Rickettsia* spp.

*Rickettsia* spp. DNA was detected in 204/1261 fleas (16.2%, 95% CI 14.3–18.3) (Table 2). The highest prevalence was observed in *C. felis* (16.8%, 95% CI 14.8–19.1) and in fleas collected from the AML region (26.0%, 95% CI 18.3–35.6), although the largest number of *Rickettsia* spp.-positive fleas was recovered from the Norte region (*n* = 62; 13.2%, 95% CI 10.4–16.5).

The fitted multivariable logistic regression model identified flea species, host species and geographic region as independent predictors of *Rickettsia* spp. positivity (*G*^2^ = 21.71, *df* = 6, *P* = 0.001) (Additional file 2: Table S2). *Ctenocephalides felis* fleas showed higher odds of *Rickettsia* spp. positivity than fleas of other species (aOR = 3.59, 95% CI 1.28–10.09; Wald* χ*^2^ = 5.88, *df* = 1, *P* = 0.015), while fleas from cats showed slightly lower odds compared with those from dogs (aOR = 0.70, 95% CI 0.51–0.95, Wald* χ*^2^ = 5.27, *df* = 1, *P* = 0.022). Significantly lower odds of positivity were observed in the Norte (aOR = 0.44, 95% CI 0.26–0.75; Wald* χ*^2^ = 9.15, *df* = 1, *P* = 0.002) and Algarve (aOR = 0.51, 95% CI 0.29–0.91; Wald* χ*^2^ = 5.17, *df* = 1, *P* = 0.023) regions compared with AML.

**Fig. 3:**
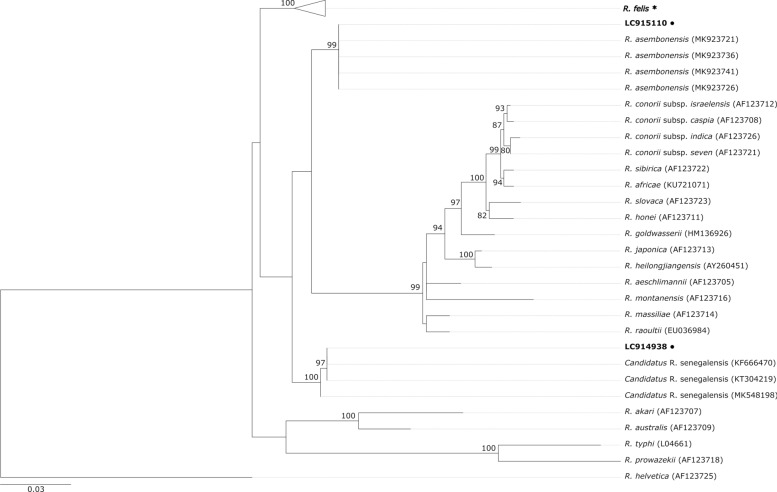
Maximum likelihood phylogenetic tree inferred from the outer membrane protein B gene sequences of *Rickettsia* spp. detected in fleas collected from dogs and cats in Portugal. Tree reconstruction was performed in IQ-TREE using the best-fitting nucleotide substitution model selected according to the Bayesian Information Criterion. Node support was assessed using 1000 bootstrap replicates, and values ≥ 75% are shown at the corresponding nodes. Reference sequences are labeled with species designation and GenBank accession numbers. Sequences obtained in this study are indicated in bold by their respective GenBank accession numbers; circle symbols denote sequences obtained from *Ctenocephalides felis*. Owing to the large number of sequences included, the *R. felis* cluster was collapsed and is indicated in bold and by an asterisk. The complete tree with the *R. felis* cluster expanded is provided in Additional file 4: Fig. S1. Associated sequence metadata are provided in Additional file 1: Tables S2. Branch lengths are scaled to the number of substitutions per site

Most sequences (*n* = 192) clustered into a stable, well-supported monophyletic clade with reference sequences of *R. felis*. Phylogenetic analysis further supported the detection of *R. asembonensis* in a *C. felis* specimen from a dog in the Centro region and *Candidatus* R. senegalensis in a *C. felis* specimen from a cat in the Algarve region (Fig. 3; Table 3; Additional file 4: Fig. S1). The remaining 10 sequences were confirmed as *Rickettsia* spp. by BLASTn analysis, with the majority showing highest nucleotide identity to *R. felis* reference sequences, but were excluded from phylogenetic analysis as their inclusion compromised phylogenetic resolution.

### *Dipylidium caninum*

*Dipylidium caninum* DNA was detected in 23/1261 fleas (1.8%; 95% CI 1.2–2.7). All positive fleas were *C. felis* (1.9%; 95% CI 1.3–2.9), and were collected from both dogs and cats (Table 2). No significant associations were observed with any studied variables, and no predictors were retained in the multivariable logistic regression model.

**Fig. 4:**
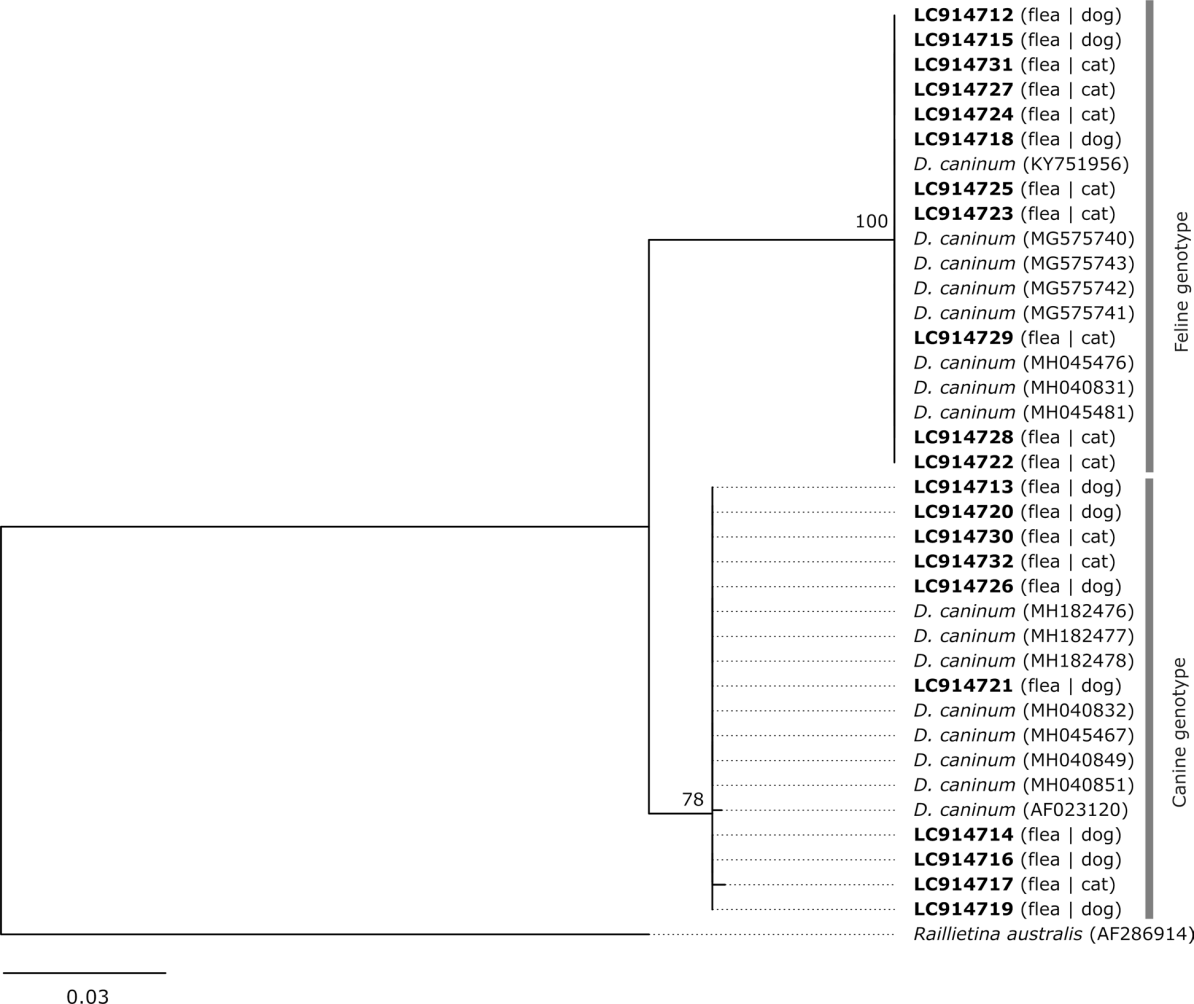
Maximum likelihood phylogenetic tree inferred from 28S rRNA gene sequences of *Dipylidium caninum* detected in *Ctenocephalides felis* collected from dogs and cats in Portugal. Tree reconstruction was performed in IQ-TREE using the best-fitting nucleotide substitution model selected according to the Bayesian Information Criterion. Node support was assessed using 1000 bootstrap replicates, and values ≥ 75% are shown at the corresponding nodes. The tree was rooted using a *Raillietina australis* (GenBank accession no. AF286914) sequence as outgroup. Reference sequences are labeled with species designation and GenBank accession numbers. Sequences obtained in this study are indicated in bold by their respective GenBank accession numbers. Associated sequence metadata are provided in Additional file 1: Table S3. Branch lengths are scaled to the number of substitutions per site

Phylogenetic analysis showed that, among the sequences included, 11 clustered exclusively with reference sequences of the feline-associated genotype, whereas 10 grouped within the canine-associated genotype (Fig. 4). Both feline and canine genotypes were detected in fleas collected from dogs and cats. The feline genotype was more frequently detected in fleas from cats (8/11, 72.7%), whereas the canine genotype was more frequently detected in fleas from dogs (7/10, 70.0%).

Although genotypic assignment was not possible for the remaining two *D. caninum*-positive fleas, their sequences showed 100% nucleotide identity and query coverage with *D. caninum* reference sequences (e.g., GenBank accession MG575740) in BLASTn analysis.

### Co-detection of pathogen DNA

DNA from more than one pathogen was detected in 2.1% (27/1261) of analyzed fleas (Table 3). Co-detection of *Bartonella* spp. and *Rickettsia* spp. was the most frequent combination (*n* = 23, 85.2%), followed by *Rickettsia* spp. and *D. caninum* (*n* = 2, 7.4%), and *Bartonella* spp. and *D. caninum* (*n* = 2, 7.4%). No flea tested positive for all three pathogens. Co-detections occurred almost exclusively in *C. felis* (26/27, 96.3%), with only one case in *P. irritans*; no co-detections were identified in *C. canis.*

## Discussion

To the best of our knowledge, the present study represents the most extensive nationwide molecular assessment of *Bartonella* spp., *Rickettsia* spp., and *D. caninum* in fleas collected from dogs and cats across mainland Portugal. The results provide updated evidence on the circulation of flea-borne pathogens with zoonotic relevance at the individual vector level and underscore the importance of flea surveillance and flea control measures within a One Health framework.

Overall, DNA of at least one target pathogen was detected in nearly one quarter of analyzed fleas, indicating ongoing circulation of zoonotic agents within flea populations parasitizing companion animals in Portugal. Most pathogen DNA-positive specimens corresponded to *C. felis*, consistent with its predominance among the fleas sampled (94.2%). Although molecular detection does not demonstrate vector competence or active transmission, identification of pathogen DNA in fleas provides a reliable framework for mapping pathogen circulation and distribution, helping to identify exposure risks and guide surveillance and preventive strategies in environments where pets and humans are in close contact [3, 9, 44].

*Bartonella* spp. DNA was detected in 8.3% of analyzed fleas, a prevalence comparable to those reported in previous studies in Europe, including Spain [10] and the UK [3]. The significantly higher prevalence observed in fleas collected from cats, particularly in stray and shelter animals, is consistent with the recognized role of cats as the primary reservoir hosts of *B. henselae* and *B. clarridgeae* [20]. Recent molecular data from Portugal further support endemic circulation of *B. henselae* in apparently healthy cats [45], reinforcing the importance of felines in sustaining flea-borne zoonotic transmission cycles, especially in populations characterized by limited veterinary care, irregular ectoparasite control, and high-density living conditions [13]. By contrast, the absence of positive fleas from strictly indoor animals aligns with evidence that restricted outdoor access reduces exposure to infected fleas and the risk of zoonotic transmission [46].

The higher proportion of *Bartonella* spp.-positive fleas in AML should be interpreted in light of nationwide infestation data. Pereira et al. [1] reported significantly lower odds of flea infestation in AML compared with the Norte region. Therefore, the results observed here likely reflect differences in host population structure rather than infestation pressure, potentially including higher numbers of stray animals, shelter populations, or multi-cat households, conditions previously associated with increased *Bartonella* spp. circulation [13, 47, 48].

*Bartonella clarridgeiae* and *B. henselae* were the species most frequently detected among analyzed fleas, predominantly *C. felis*. These findings are consistent with multiple studies and reinforce the role of *C. felis* as the principal vector of these zoonotic pathogens worldwide [4, 5]. *Bartonella henselae* is the main etiological agent of cat-scratch disease and can also lead to bacillary angiomatosis, neuroretinitis, and endocarditis, particularly in immunocompromised patients, reinforcing the public health importance of effective flea control in companion animals [49, 50]. In Portugal, these species have also been detected in cats and their fleas [37, 48], and several human cases of *Bartonella* spp. infection, including typical and atypical cat-scratch disease presentations, have been reported [51–54], supporting the local relevance of these findings.

By contrast, identification of *B. rochalimae* DNA in 3 fleas represents, to our knowledge, the first report of this emerging zoonotic species in Portugal. Its detection in *P. irritans* further supports the potential role of the so‑called human flea as an ecologically relevant vector [55]. Detection in companion animal fleas suggests that this bacterium circulates locally, although additional investigations in domestic and wild hosts are required to clarify its epidemiological relevance.

*Rickettsia* spp. DNA was detected in approximately one in six analyzed fleas, indicating substantial circulation of these pathogens among fleas infesting companion animals in Portugal. Most positive specimens corresponded to *C. felis*, with sequence analysis revealing predominance of *R. felis*. These findings are consistent with extensive evidence identifying *C. felis* as both the primary vector and invertebrate reservoir host of *R. felis*, capable of maintaining infection through efficient vertical and horizontal transmission within flea populations [56–58].

From a public health perspective, *R. felis* causes flea‑borne spotted fever in humans, typically presenting as a nonspecific febrile illness that is likely underdiagnosed due to clinical overlap with other febrile syndromes [59]. The widespread detection of infected fleas in domestic environments therefore indicates a persistent, often underappreciated pathway for human exposure, reinforcing the importance of effective flea control in integrated prevention strategies [60].

Multivariable analyses indicated that odds of pathogen presence were higher in fleas collected from dogs than from cats, supporting previous evidence identifying dogs as reservoir hosts of *R. felis* [25, 61]. Experimental and field data indicate that dogs can develop prolonged, frequently subclinical rickettsemia and can infect naïve fleas [25, 60]. Given their close contact with humans and their role in sustaining flea populations, dogs may facilitate maintenance of domestic and peridomestic transmission cycles and increase risk of human exposure [25, 61, 62].

Detection of *R. asembonensis* and *Ca.* R. senegalensis expands the known diversity of flea-associated rickettsiae circulating in Portugal. *Rickettsia asembonensis* have been increasingly reported worldwide in fleas associated with domestic and wild hosts and has occasionally been linked to human febrile illness [29, 63]. In Portugal, the pathogen was previously detected in *A. erinacei* specimens from rescued European hedgehogs, suggesting a local enzootic cycle [26]. In this study, the detection of *R. asembonensis* in *C. felis* from a pet dog suggests that this flea-borne pathogen is not restricted to wildlife-associated cycles.

Regarding *Ca.* R. senegalensis, its detection in *C. felis* from a cat represents, to our knowledge, the first report of this species in Europe, thereby extending its known geographical range beyond Africa, Asia, and the Americas [64–70]. Possible, nonexclusive hypotheses explaining its detection include international movement of pets, cross-border movements of stray or shelter animals, or natural dispersal of wild or synanthropic hosts carrying infected fleas. These remain speculative and require targeted, longitudinal surveillance studies. Although its pathogenicity to humans has not been established, the presence of this pathogen in a widespread human-biting flea suggests potential exposure at the human-animal interface [70].

The prevalence of *D. caninum* DNA was low (1.8%), similar to those reported in other PCR-based flea surveys in European countries, including the UK (3.0%) [3], France (1.1%), Germany (1.4%), Slovenia (1.3%), and the Czech Republic (3.1%), but markedly lower than that reported in Romania (16.7%) [39]. This suggests that even under substantial flea infestation pressure, only a small proportion of fleas harbor infective cysticercoids at any given time, helping to explain the relatively low prevalence of *D. caninum* infection among canine and feline populations relative to other common parasitic infections [11, 71].

All *D. caninum*-positive specimens belonged to *C. felis*, corroborating its role as the main intermediate host worldwide [11]. Absence of significant associations with host or environmental variables suggests relatively homogeneous exposure among infested animals [3]. Nevertheless, even low vector infection prevalence implies cumulative exposure risk without sustained ectoparasite control [11]. Notably, control and prevention of flea infestations alone has been shown to be efficacious in preventing *D. caninum* infection [72], potentially reducing the need for routine tapeworm-directed anthelmintic treatment.

Genotypic characterization demonstrated circulation of both canine- and feline-associated *D. caninum* genotypes in fleas collected from dogs and cats [33, 39]. Previous studies show that infections are largely, although not exclusively, host-associated [33, 39]. The predominance of the feline genotype in fleas from cats and the canine genotype in fleas from dogs observed in this study mirrors previously described patterns and supports the existence of host-adapted lineages. However, the detection of both genotypes in fleas from either host does not necessarily imply cross-host species infection, potentially reflecting exposure to flea feces from animals of a different host species, particularly in multi-animal environments [11]. Alongside genomic evidence suggesting that these genotypes may represent distinct species [73], the present findings suggest that *C. felis* populations can simultaneously maintain and transmit both genotypes. This has implications for understanding local transmission dynamics and zoonotic risk, as well as for future studies assessing potential genotype-specific differences in pathogenicity and anthelmintic susceptibility [11].

Co-detection of pathogens within individual fleas, most commonly involving *Bartonella* spp. and *Rickettsia* spp., likely reflects cumulative exposure events rather than biological pathogen interactions [74]. Clarifying their ecological relevance will require longitudinal and experimental studies.

Some limitations of this study should be acknowledged. The use of pooled DNA for initial screening, with each specimen contributing 1 µl per reaction, may have reduced the sensitivity of pathogen detection compared with individual screening, potentially leading to underestimation of true infection prevalence, particularly for pathogens present at low DNA concentrations. Additionally, the cross-sectional design does not allow inference of temporal transmission dynamics or causality. Sample sizes for some flea species, namely *C. canis* (*n* = 54), *P. irritans* (*n* = 14), and *A. erinacei* (*n* = 5), were limited, restricting the statistical power of species-specific comparisons. Finally, molecular detection of pathogen DNA in fleas does not confirm viability or active transmission, nor does it provide information on infection status of the vertebrate host.

## Conclusions

This nationwide molecular survey demonstrates that fleas infesting companion animals in mainland Portugal frequently harbor pathogens of veterinary and zoonotic relevance, including *Bartonella* spp., *Rickettsia* spp., and *D. caninum*. The predominance of *C. felis* among the fleas infesting dogs and cats further highlights the central role of this species in maintaining and potentially facilitating pathogen circulation at the animal–human interface. Associations observed with host species, lifestyle, and geographic region indicate that pathogen occurrence is shaped by local ecological and host-related factors.

The detection of zoonotic and emerging agents, including species not previously reported in Portugal, reinforces the need to recognize flea infestations as both a veterinary and public health concern. Continued molecular surveillance integrating vector, host, and environmental data will be essential to clarify transmission dynamics and support evidence-based control strategies aimed at reducing pathogen circulation and zoonotic exposure within a One Health framework.

## Supplementary Information

Supplementary Material 1.

Supplementary Material 2.

Supplementary Material 3 (Additional file 3: Fig. S1 Complete maximum likelihood phylogenetic tree inferred from 16S–23S rRNA intergenic spacer region sequences of *Bartonella *spp. detected in fleas collected from dogs and cats in Portugal, including all study sequences. Tree reconstruction was performed in IQ-TREE using the best-fitting nucleotide substitution model selected according to the Bayesian Information Criterion. Node support was assessed using 1000 bootstrap replicates, and values ≥75% are shown at the corresponding nodes. Reference sequences are labeled with species designation and GenBank accession numbers. Sequences obtained in this study are indicated in bold by their respective GenBank accession numbers; Circle symbols denote sequences obtained from *Ctenocephalides felis*, square symbols represent sequences obtained from Ctenocephalides canis, and triangle symbols indicate sequences obtained from *Pulex irritans*. Associated sequence metadata are provided in Additional file 1: Tables S1. Branch lengths are scaled to the number of substitutions per site.)

Supplementary Material 4 (Additional file 4: Fig. S1 Complete maximum likelihood phylogenetic tree inferred from the outer membrane protein B gene sequences of *Rickettsia* spp. detected in fleas collected from dogs and cats in Portugal, including all study sequences. Tree reconstruction was performed in IQ-TREE using the best-fitting nucleotide substitution model selected according to the Bayesian Information Criterion. Node support was assessed using 1000 bootstrap replicates, and values ≥75% are shown at the corresponding nodes. Reference sequences are labeled with species designation and GenBank accession numbers. Sequences obtained in this study are indicated in bold by their respective GenBank accession numbers; circle symbols denote sequences obtained from *Ctenocephalides felis*, whereas triangle symbols indicate sequences obtained from *Pulex irritans*. Associated sequence metadata are provided in Additional file 1: Tables S2. Branch lengths are scaled to the number of substitutions per site.)

## Data Availability

All data supporting the findings of this study are available within the manuscript and its Supplementary Information.

## Abbreviations

AML: Área Metropolitana de Lisboa
ASR: Adjusted standardised residuals
AUC: Area under the receiver operating characteristic curve
aOR: Adjusted odds ratio
BLASTn: Basic local alignment search tool
CI: Confidence interval
GISCO: Geographic Information System of the European Commission
NUTS II: Nomenclature of Units for Territorial Statistics II
PCR: Polymerase chain reaction
*ompB*: Outer membrane protein B
rRNA: Ribosomal RNA
