# Supplementary material for: Molecular detection of zoonotic pathogens in fleas collected from dogs and cats in Portugal
Source: Parasit Vectors. 2026 May 30;19:311. doi: 10.1186/s13071-026-07456-4 (PMC13435523; doi:10.1186/s13071-026-07456-4)
Supplement: Supplementary file 1 [file 13071_2026_7456_MOESM1_ESM.docx]

**Table S1**. Metadata of Bartonella spp. 16S-23S intergenic spacer region sequences included in the phylogenetic analysis.

| Species | Invertebrate host | Vertebrate host | Country | Accession number |
| --- | --- | --- | --- | --- |
| *Bartonella clarridgeiae* | *Ctenocephalides felis* | *Felis catus* | Portugal | LC914763* |
| *Bartonella clarridgeiae* | *Ctenocephalides felis* | *Felis catus* | Portugal | LC914764* |
| *Bartonella clarridgeiae* | *Ctenocephalides felis* | *Felis catus* | Portugal | LC914765* |
| *Bartonella clarridgeiae* | *Ctenocephalides felis* | *Felis catus* | Portugal | LC914766* |
| *Bartonella clarridgeiae* | *Ctenocephalides felis* | *Felis catus* | Portugal | LC914767* |
| *Bartonella henselae* | *Ctenocephalides felis* | *Felis catus* | Portugal | LC914768* |
| *Bartonella clarridgeiae* | *Ctenocephalides felis* | *Felis catus* | Portugal | LC914769* |
| *Bartonella henselae* | *Ctenocephalides felis* | *Canis lupus familiaris* | Portugal | LC914770* |
| *Bartonella clarridgeiae* | *Ctenocephalides felis* | *Felis catus* | Portugal | LC914771* |
| *Bartonella clarridgeiae* | *Ctenocephalides felis* | *Felis catus* | Portugal | LC914772* |
| *Bartonella clarridgeiae* | *Ctenocephalides felis* | *Canis lupus familiaris* | Portugal | LC914773* |
| *Bartonella henselae* | *Ctenocephalides felis* | *Felis catus* | Portugal | LC914774* |
| *Bartonella clarridgeiae* | *Ctenocephalides felis* | *Felis catus* | Portugal | LC914775* |
| *Bartonella henselae* | *Ctenocephalides felis* | *Felis catus* | Portugal | LC914776* |
| *Bartonella clarridgeiae* | *Ctenocephalides felis* | *Felis catus* | Portugal | LC914777* |
| *Bartonella henselae* | *Ctenocephalides felis* | *Canis lupus familiaris* | Portugal | LC914778* |
| *Bartonella clarridgeiae* | *Ctenocephalides felis* | *Felis catus* | Portugal | LC914779* |
| *Bartonella clarridgeiae* | *Ctenocephalides felis* | *Canis lupus familiaris* | Portugal | LC914780* |
| *Bartonella clarridgeiae* | *Ctenocephalides felis* | *Canis lupus familiaris* | Portugal | LC914781* |
| *Bartonella clarridgeiae* | *Ctenocephalides felis* | *Canis lupus familiaris* | Portugal | LC914782* |
| *Bartonella henselae* | *Ctenocephalides felis* | *Felis catus* | Portugal | LC914783* |
| *Bartonella henselae* | *Ctenocephalides felis* | *Felis catus* | Portugal | LC914784* |
| *Bartonella henselae* | *Ctenocephalides felis* | *Felis catus* | Portugal | LC914785* |
| *Bartonella henselae* | *Ctenocephalides felis* | *Felis catus* | Portugal | LC914786* |
| *Bartonella henselae* | *Ctenocephalides felis* | *Felis catus* | Portugal | LC914787* |
| *Bartonella henselae* | *Ctenocephalides felis* | *Felis catus* | Portugal | LC914788* |
| *Bartonella henselae* | *Ctenocephalides felis* | *Felis catus* | Portugal | LC914789* |
| *Bartonella clarridgeiae* | *Ctenocephalides felis* | *Felis catus* | Portugal | LC914790* |
| *Bartonella clarridgeiae* | *Ctenocephalides felis* | *Felis catus* | Portugal | LC914791* |
| *Bartonella clarridgeiae* | *Ctenocephalides felis* | *Felis catus* | Portugal | LC914792* |
| *Bartonella clarridgeiae* | *Ctenocephalides felis* | *Felis catus* | Portugal | LC914793* |
| *Bartonella clarridgeiae* | *Ctenocephalides felis* | *Felis catus* | Portugal | LC914794* |
| *Bartonella clarridgeiae* | *Ctenocephalides felis* | *Felis catus* | Portugal | LC914795* |
| *Bartonella henselae* | *Ctenocephalides felis* | *Felis catus* | Portugal | LC914796* |
| *Bartonella henselae* | *Ctenocephalides felis* | *Felis catus* | Portugal | LC914797* |
| *Bartonella clarridgeiae* | *Ctenocephalides felis* | *Felis catus* | Portugal | LC914798* |
| *Bartonella clarridgeiae* | *Ctenocephalides felis* | *Felis catus* | Portugal | LC914799* |
| *Bartonella henselae* | *Ctenocephalides felis* | *Canis lupus familiaris* | Portugal | LC914800* |
| *Bartonella rochalimae* | *Pulex irritans* | *Canis lupus familiaris* | Portugal | LC914801* |
| *Bartonella clarridgeiae* | *Ctenocephalides felis* | *Felis catus* | Portugal | LC914802* |
| *Bartonella clarridgeiae* | *Ctenocephalides felis* | *Felis catus* | Portugal | LC914803* |
| *Bartonella clarridgeiae* | *Ctenocephalides felis* | *Felis catus* | Portugal | LC914804* |
| *Bartonella henselae* | *Ctenocephalides felis* | *Felis catus* | Portugal | LC914805* |
| *Bartonella clarridgeiae* | *Ctenocephalides felis* | *Felis catus* | Portugal | LC914806* |
| *Bartonella clarridgeiae* | *Ctenocephalides felis* | *Felis catus* | Portugal | LC914807* |
| *Bartonella clarridgeiae* | *Ctenocephalides felis* | *Felis catus* | Portugal | LC914808* |
| *Bartonella clarridgeiae* | *Ctenocephalides felis* | *Felis catus* | Portugal | LC914809* |
| *Bartonella clarridgeiae* | *Ctenocephalides felis* | *Felis catus* | Portugal | LC914810* |
| *Bartonella henselae* | *Ctenocephalides felis* | *Felis catus* | Portugal | LC914811* |
| *Bartonella henselae* | *Ctenocephalides felis* | *Felis catus* | Portugal | LC914812* |
| *Bartonella henselae* | *Ctenocephalides felis* | *Felis catus* | Portugal | LC914813* |
| *Bartonella henselae* | *Ctenocephalides felis* | *Felis catus* | Portugal | LC914814* |
| *Bartonella clarridgeiae* | *Ctenocephalides felis* | *Felis catus* | Portugal | LC914815* |
| *Bartonella clarridgeiae* | *Ctenocephalides felis* | *Felis catus* | Portugal | LC914816* |
| *Bartonella henselae* | *Ctenocephalides felis* | *Felis catus* | Portugal | LC914817* |
| *Bartonella clarridgeiae* | *Ctenocephalides felis* | *Felis catus* | Portugal | LC914818* |
| *Bartonella clarridgeiae* | *Ctenocephalides felis* | *Felis catus* | Portugal | LC914819* |
| *Bartonella clarridgeiae* | *Ctenocephalides felis* | *Felis catus* | Portugal | LC914820* |
| *Bartonella clarridgeiae* | *Ctenocephalides felis* | *Felis catus* | Portugal | LC914821* |
| *Bartonella henselae* | *Ctenocephalides felis* | *Felis catus* | Portugal | LC914822* |
| *Bartonella clarridgeiae* | *Ctenocephalides felis* | *Felis catus* | Portugal | LC914823* |
| *Bartonella henselae* | *Ctenocephalides felis* | *Felis catus* | Portugal | LC914824* |
| *Bartonella clarridgeiae* | *Ctenocephalides felis* | *Felis catus* | Portugal | LC914825* |
| *Bartonella clarridgeiae* | *Ctenocephalides felis* | *Felis catus* | Portugal | LC914826* |
| *Bartonella clarridgeiae* | *Ctenocephalides felis* | *Felis catus* | Portugal | LC914827* |
| *Bartonella henselae* | *Ctenocephalides felis* | *Felis catus* | Portugal | LC914828* |
| *Bartonella clarridgeiae* | *Ctenocephalides felis* | *Felis catus* | Portugal | LC914829* |
| *Bartonella henselae* | *Ctenocephalides felis* | *Felis catus* | Portugal | LC914830* |
| *Bartonella clarridgeiae* | *Ctenocephalides felis* | *Felis catus* | Portugal | LC914831* |
| *Bartonella clarridgeiae* | *Ctenocephalides felis* | *Felis catus* | Portugal | LC914832* |
| *Bartonella clarridgeiae* | *Ctenocephalides felis* | *Felis catus* | Portugal | LC914833* |
| *Bartonella henselae* | *Ctenocephalides felis* | *Felis catus* | Portugal | LC914834* |
| *Bartonella rochalimae* | *Ctenocephalides felis* | *Canis lupus familiaris* | Portugal | LC914835* |
| *Bartonella henselae* | *Ctenocephalides felis* | *Felis catus* | Portugal | LC914836* |
| *Bartonella clarridgeiae* | *Ctenocephalides felis* | *Canis lupus familiaris* | Portugal | LC914837* |
| *Bartonella henselae* | *Ctenocephalides canis* | *Canis lupus familiaris* | Portugal | LC914838* |
| *Bartonella henselae* | *Ctenocephalides felis* | *Felis catus* | Portugal | LC914839* |
| *Bartonella rochalimae* | *Pulex irritans* | *Canis lupus familiaris* | Portugal | LC914840* |
| *Bartonella henselae* | *Ctenocephalides felis* | *Felis catus* | Portugal | LC914841* |
| *Bartonella henselae* | *Ctenocephalides felis* | *Felis catus* | Portugal | LC914842* |
| *Bartonella clarridgeiae* | *Ctenocephalides felis* | *Felis catus* | Portugal | LC914843* |
| *Bartonella henselae* | *Ctenocephalides felis* | *Felis catus* | Portugal | LC914844* |
| *Bartonella clarridgeiae* | *Ctenocephalides felis* | *Felis catus* | Portugal | LC914845* |
| *Bartonella henselae* | *Ctenocephalides felis* | *Felis catus* | Portugal | LC914846* |
| *Bartonella henselae* | *Ctenocephalides felis* | *Felis catus* | Portugal | LC914847* |
| *Bartonella clarridgeiae* | *Ctenocephalides felis* | *Felis catus* | Portugal | LC914848* |
| *Bartonella clarridgeiae* | *Ctenocephalides felis* | *Felis catus* | Portugal | LC914849* |
| *Bartonella clarridgeiae* | *Ctenocephalides felis* | *Felis catus* | Portugal | LC914850* |
| *Bartonella henselae* | *Ctenocephalides felis* | *Felis catus* | Portugal | LC914851* |
| *Bartonella clarridgeiae* | *Ctenocephalides felis* | *Felis catus* | Portugal | LC914852* |
| *Bartonella henselae* | *Ctenocephalides felis* | *Canis lupus familiaris* | Portugal | LC914853* |
| *Bartonella henselae* | *Ctenocephalides felis* | *Felis catus* | Portugal | LC914854* |
| *Bartonella clarridgeiae* | *Ctenocephalides felis* | *Felis catus* | Portugal | LC914855* |
| *Bartonella henselae* | *Ctenocephalides felis* | *Felis catus* | Portugal | LC914856* |
| *Bartonella clarridgeiae* | *Ctenocephalides felis* | *Felis catus* | Portugal | LC914857* |
| *Bartonella henselae* | *Ctenocephalides felis* | *Felis catus* | Portugal | LC914858* |
| *Bartonella clarridgeiae* | *Ctenocephalides felis* | *Felis catus* | Portugal | LC914859* |
| *Bartonella clarridgeiae* | *Ctenocephalides felis* | *Felis catus* | Portugal | LC914860* |
| *Bartonella clarridgeiae* | *Ctenocephalides felis* | *Felis catus* | Portugal | LC914861* |
| *Bartonella clarridgeiae* | *Ctenocephalides felis* | *Felis catus* | Portugal | LC914862* |
| *Bartonella clarridgeiae* | *Ctenocephalides felis* | *Felis catus* | Portugal | LC914863* |
| *Bartonella clarridgeiae* | *Ctenocephalides felis* | *Felis catus* | Portugal | LC914864* |
| *Bartonella clarridgeiae* | *Ctenocephalides felis* | *Canis lupus familiaris* | Portugal | LC914865* |
| *Bartonella clarridgeiae* | *Ctenocephalides felis* | *Felis catus* | Portugal | LC914866* |
| *Bartonella henselae* | NA | *Felis catus* | NA | MZ323357 |
| *Bartonella vinsonii* subsp*. vinsonii* | NA | NA | NA | L35102 |
| *Bartonella vinsonii* subsp*. berkhoffi* | NA | NA | NA | DQ059765 |
| *Bartonella vinsonii* subsp*. berkhoffi* | NA | NA | NA | DQ059764 |
| *Bartonella vinsonii* subsp*. berkhoffi* | NA | NA | NA | DQ059763 |
| *Bartonella elizabethae* | *Leptopsylla taschenbergi* | NA | France | LR746190 |
| *Bartonella elizabethae* | *Stenoponia tripectinata tripectinata* | NA | France | LR746177 |
| *Bartonella elizabethae* | NA | *Meriones libycus* | Georgia | JF766264 |
| *Bartonella tribocorum* | NA | NA | NA | AF312505 |
| *Bartonella grahamii* | NA | *Apodemus agrarius* | South Korea | JN810847 |
| *Bartonella tribocorum* | NA | *Apodemus agrarius* | Lithuania | MH687379 |
| *Bartonella rochalimae* | NA | NA | NA | DQ676491 |
| *Bartonella rochalimae* | NA | *Procyon lotor* | Germany | PQ497332 |
| *Bartonella rochalimae* | NA | *Procyon lotor* | USA | KU292577 |
| *Bartonella clarridgeiae* | NA | NA | NA | AF312497 |
| *Bartonella clarridgeiae* | NA | *Felis catus* | USA | DQ683194 |
| *Bartonella clarridgeiae* | NA | NA | NA | AF167989 |
| *Bartonella clarridgeiae* | NA | NA | NA | FN645454 |

***** Sequences generated in the present study; all other sequences were retrieved from GenBank.

NA, not available.

**Table S2.** Metadata of Rickettsia spp. outer membrane protein B (ompB) sequences included in the phylogenetic analysis.

| Species | Invertebrate host | Vertebrate host | Country | Accession number |
| --- | --- | --- | --- | --- |
| *Rickettsia felis* | *Ctenocephalides felis* | *Felis catus* | Portugal | LC914867* |
| *Rickettsia felis* | *Ctenocephalides felis* | *Felis catus* | Portugal | LC914868* |
| *Rickettsia felis* | *Ctenocephalides felis* | *Felis catus* | Portugal | LC914869* |
| *Rickettsia felis* | *Ctenocephalides felis* | *Canis lupus familiaris* | Portugal | LC914870* |
| *Rickettsia felis* | *Ctenocephalides felis* | *Canis lupus familiaris* | Portugal | LC914871* |
| *Rickettsia felis* | *Ctenocephalides felis* | *Canis lupus familiaris* | Portugal | LC914872* |
| *Rickettsia felis* | *Ctenocephalides felis* | *Felis catus* | Portugal | LC914873* |
| *Rickettsia felis* | *Ctenocephalides felis* | *Canis lupus familiaris* | Portugal | LC914874* |
| *Rickettsia felis* | *Ctenocephalides felis* | *Felis catus* | Portugal | LC914875* |
| *Rickettsia felis* | *Ctenocephalides felis* | *Felis catus* | Portugal | LC914876* |
| *Rickettsia felis* | *Ctenocephalides felis* | *Felis catus* | Portugal | LC914877* |
| *Rickettsia felis* | *Ctenocephalides felis* | *Felis catus* | Portugal | LC914878* |
| *Rickettsia felis* | *Ctenocephalides felis* | *Canis lupus familiaris* | Portugal | LC914879* |
| *Rickettsia felis* | *Ctenocephalides felis* | *Canis lupus familiaris* | Portugal | LC914880* |
| *Rickettsia felis* | *Ctenocephalides felis* | *Felis catus* | Portugal | LC914881* |
| *Rickettsia felis* | *Ctenocephalides felis* | *Felis catus* | Portugal | LC914882* |
| *Rickettsia felis* | *Ctenocephalides felis* | *Canis lupus familiaris* | Portugal | LC914883* |
| *Rickettsia felis* | *Ctenocephalides felis* | *Canis lupus familiaris* | Portugal | LC914884* |
| *Rickettsia felis* | *Ctenocephalides felis* | *Canis lupus familiaris* | Portugal | LC914885* |
| *Rickettsia felis* | *Ctenocephalides felis* | *Canis lupus familiaris* | Portugal | LC914886* |
| *Rickettsia felis* | *Ctenocephalides felis* | *Felis catus* | Portugal | LC914887* |
| *Rickettsia felis* | *Ctenocephalides felis* | *Canis lupus familiaris* | Portugal | LC914888* |
| *Rickettsia felis* | *Ctenocephalides felis* | *Canis lupus familiaris* | Portugal | LC914889* |
| *Rickettsia felis* | *Pulex irritans* | *Canis lupus familiaris* | Portugal | LC914890* |
| *Rickettsia felis* | *Ctenocephalides felis* | *Felis catus* | Portugal | LC914891* |
| *Rickettsia felis* | *Pulex irritans* | *Canis lupus familiaris* | Portugal | LC914892* |
| *Rickettsia felis* | *Ctenocephalides felis* | *Canis lupus familiaris* | Portugal | LC914893* |
| *Rickettsia felis* | *Ctenocephalides felis* | *Felis catus* | Portugal | LC914894* |
| *Rickettsia felis* | *Ctenocephalides felis* | *Felis catus* | Portugal | LC914895* |
| *Rickettsia felis* | *Ctenocephalides felis* | *Canis lupus familiaris* | Portugal | LC914896* |
| *Rickettsia felis* | *Ctenocephalides felis* | *Canis lupus familiaris* | Portugal | LC914897* |
| *Rickettsia felis* | *Ctenocephalides felis* | *Canis lupus familiaris* | Portugal | LC914898* |
| *Rickettsia felis* | *Ctenocephalides felis* | *Felis catus* | Portugal | LC914899* |
| *Rickettsia felis* | *Ctenocephalides felis* | *Canis lupus familiaris* | Portugal | LC914900* |
| *Rickettsia felis* | *Ctenocephalides felis* | *Canis lupus familiaris* | Portugal | LC914901* |
| *Rickettsia felis* | *Ctenocephalides felis* | *Canis lupus familiaris* | Portugal | LC914902* |
| *Rickettsia felis* | *Ctenocephalides felis* | *Canis lupus familiaris* | Portugal | LC914903* |
| *Rickettsia felis* | *Ctenocephalides felis* | *Felis catus* | Portugal | LC914904* |
| *Rickettsia felis* | *Ctenocephalides felis* | *Canis lupus familiaris* | Portugal | LC914905* |
| *Rickettsia felis* | *Ctenocephalides felis* | *Felis catus* | Portugal | LC914906* |
| *Rickettsia felis* | *Ctenocephalides felis* | *Felis catus* | Portugal | LC914907* |
| *Rickettsia felis* | *Ctenocephalides felis* | *Canis lupus familiaris* | Portugal | LC914908* |
| *Rickettsia felis* | *Ctenocephalides felis* | *Canis lupus familiaris* | Portugal | LC914909* |
| *Rickettsia felis* | *Ctenocephalides felis* | *Canis lupus familiaris* | Portugal | LC914910* |
| *Rickettsia felis* | *Ctenocephalides felis* | *Canis lupus familiaris* | Portugal | LC914911* |
| *Rickettsia felis* | *Ctenocephalides felis* | *Felis catus* | Portugal | LC914912* |
| *Rickettsia felis* | *Ctenocephalides felis* | *Felis catus* | Portugal | LC914913* |
| *Rickettsia felis* | *Ctenocephalides felis* | *Felis catus* | Portugal | LC914914* |
| *Rickettsia felis* | *Ctenocephalides felis* | *Felis catus* | Portugal | LC914915* |
| *Rickettsia felis* | *Ctenocephalides felis* | *Felis catus* | Portugal | LC914916* |
| *Rickettsia felis* | *Ctenocephalides canis* | *Canis lupus familiaris* | Portugal | LC914917* |
| *Rickettsia felis* | *Ctenocephalides felis* | *Felis catus* | Portugal | LC914918* |
| *Rickettsia felis* | *Ctenocephalides felis* | *Felis catus* | Portugal | LC914919* |
| *Rickettsia felis* | *Ctenocephalides felis* | *Canis lupus familiaris* | Portugal | LC914920* |
| *Rickettsia felis* | *Ctenocephalides felis* | *Felis catus* | Portugal | LC914921* |
| *Rickettsia felis* | *Ctenocephalides felis* | *Felis catus* | Portugal | LC914922* |
| *Rickettsia felis* | *Ctenocephalides felis* | *Canis lupus familiaris* | Portugal | LC914923* |
| *Rickettsia felis* | *Ctenocephalides felis* | *Felis catus* | Portugal | LC914924* |
| *Rickettsia felis* | *Ctenocephalides felis* | *Canis lupus familiaris* | Portugal | LC914925* |
| *Rickettsia felis* | *Ctenocephalides felis* | *Felis catus* | Portugal | LC914926* |
| *Rickettsia felis* | *Ctenocephalides felis* | *Canis lupus familiaris* | Portugal | LC914927* |
| *Rickettsia felis* | *Ctenocephalides felis* | *Felis catus* | Portugal | LC914928* |
| *Rickettsia felis* | *Ctenocephalides felis* | *Canis lupus familiaris* | Portugal | LC914929* |
| *Rickettsia felis* | *Ctenocephalides felis* | *Canis lupus familiaris* | Portugal | LC914930* |
| *Rickettsia felis* | *Ctenocephalides felis* | *Canis lupus familiaris* | Portugal | LC914931* |
| *Rickettsia felis* | *Ctenocephalides felis* | *Felis catus* | Portugal | LC914932* |
| *Rickettsia felis* | *Ctenocephalides felis* | *Canis lupus familiaris* | Portugal | LC914933* |
| *Rickettsia felis* | *Ctenocephalides felis* | *Canis lupus familiaris* | Portugal | LC914934* |
| *Rickettsia felis* | *Ctenocephalides felis* | *Canis lupus familiaris* | Portugal | LC914935* |
| *Rickettsia felis* | *Ctenocephalides felis* | *Felis catus* | Portugal | LC914936* |
| *Rickettsia felis* | *Ctenocephalides felis* | *Felis catus* | Portugal | LC914937* |
| *Candidatus* Rickettsia senegalensis | *Ctenocephalides felis* | *Felis catus* | Portugal | LC914938* |
| *Rickettsia felis* | *Ctenocephalides felis* | *Canis lupus familiaris* | Portugal | LC914939* |
| *Rickettsia felis* | *Ctenocephalides felis* | *Canis lupus familiaris* | Portugal | LC914940* |
| *Rickettsia felis* | *Ctenocephalides felis* | *Canis lupus familiaris* | Portugal | LC914941* |
| *Rickettsia felis* | *Ctenocephalides felis* | *Felis catus* | Portugal | LC914942* |
| *Rickettsia felis* | *Ctenocephalides felis* | *Felis catus* | Portugal | LC914943* |
| *Rickettsia felis* | *Ctenocephalides felis* | *Canis lupus familiaris* | Portugal | LC914944* |
| *Rickettsia felis* | *Ctenocephalides felis* | *Canis lupus familiaris* | Portugal | LC914945* |
| *Rickettsia felis* | *Ctenocephalides felis* | *Felis catus* | Portugal | LC914946* |
| *Rickettsia felis* | *Ctenocephalides felis* | *Canis lupus familiaris* | Portugal | LC914947* |
| *Rickettsia felis* | *Ctenocephalides felis* | *Canis lupus familiaris* | Portugal | LC914948* |
| *Rickettsia felis* | *Ctenocephalides felis* | *Canis lupus familiaris* | Portugal | LC914949* |
| *Rickettsia felis* | *Ctenocephalides felis* | *Canis lupus familiaris* | Portugal | LC914950* |
| *Rickettsia felis* | *Ctenocephalides felis* | *Canis lupus familiaris* | Portugal | LC914951* |
| *Rickettsia felis* | *Ctenocephalides felis* | *Canis lupus familiaris* | Portugal | LC914952* |
| *Rickettsia felis* | *Ctenocephalides felis* | *Felis catus* | Portugal | LC914953* |
| *Rickettsia felis* | *Ctenocephalides felis* | *Canis lupus familiaris* | Portugal | LC914954* |
| *Rickettsia felis* | *Ctenocephalides felis* | *Felis catus* | Portugal | LC914955* |
| *Rickettsia felis* | *Ctenocephalides felis* | *Canis lupus familiaris* | Portugal | LC914956* |
| *Rickettsia felis* | *Ctenocephalides felis* | *Canis lupus familiaris* | Portugal | LC914957* |
| *Rickettsia felis* | *Ctenocephalides felis* | *Canis lupus familiaris* | Portugal | LC914958* |
| *Rickettsia felis* | *Ctenocephalides felis* | *Canis lupus familiaris* | Portugal | LC914959* |
| *Rickettsia felis* | *Ctenocephalides felis* | *Felis catus* | Portugal | LC914960* |
| *Rickettsia felis* | *Ctenocephalides felis* | *Felis catus* | Portugal | LC914961* |
| *Rickettsia felis* | *Ctenocephalides felis* | *Felis catus* | Portugal | LC914962* |
| *Rickettsia felis* | *Ctenocephalides felis* | *Canis lupus familiaris* | Portugal | LC914963* |
| *Rickettsia felis* | *Ctenocephalides felis* | *Canis lupus familiaris* | Portugal | LC914964* |
| *Rickettsia felis* | *Ctenocephalides felis* | *Felis catus* | Portugal | LC914965* |
| *Rickettsia felis* | *Ctenocephalides felis* | *Canis lupus familiaris* | Portugal | LC914966* |
| *Rickettsia felis* | *Ctenocephalides felis* | *Felis catus* | Portugal | LC915091* |
| *Rickettsia felis* | *Ctenocephalides felis* | *Felis catus* | Portugal | LC915092* |
| *Rickettsia felis* | *Ctenocephalides felis* | *Canis lupus familiaris* | Portugal | LC915093* |
| *Rickettsia felis* | *Ctenocephalides felis* | *Felis catus* | Portugal | LC915094* |
| *Rickettsia felis* | *Ctenocephalides felis* | *Canis lupus familiaris* | Portugal | LC915095* |
| *Rickettsia felis* | *Ctenocephalides felis* | *Canis lupus familiaris* | Portugal | LC915096* |
| *Rickettsia felis* | *Ctenocephalides felis* | *Canis lupus familiaris* | Portugal | LC915097* |
| *Rickettsia felis* | *Ctenocephalides felis* | *Felis catus* | Portugal | LC915098* |
| *Rickettsia felis* | *Ctenocephalides felis* | *Felis catus* | Portugal | LC915099* |
| *Rickettsia felis* | *Ctenocephalides felis* | *Felis catus* | Portugal | LC915100* |
| *Rickettsia felis* | *Ctenocephalides felis* | *Canis lupus familiaris* | Portugal | LC915101* |
| *Rickettsia felis* | *Ctenocephalides felis* | *Felis catus* | Portugal | LC915102* |
| *Rickettsia felis* | *Ctenocephalides felis* | *Canis lupus familiaris* | Portugal | LC915103* |
| *Rickettsia felis* | *Ctenocephalides felis* | *Felis catus* | Portugal | LC915104* |
| *Rickettsia felis* | *Ctenocephalides felis* | *Felis catus* | Portugal | LC915105* |
| *Rickettsia felis* | *Ctenocephalides felis* | *Felis catus* | Portugal | LC915106* |
| *Rickettsia felis* | *Ctenocephalides felis* | *Felis catus* | Portugal | LC915107* |
| *Rickettsia felis* | *Ctenocephalides felis* | *Canis lupus familiaris* | Portugal | LC915108* |
| *Rickettsia felis* | *Ctenocephalides felis* | *Felis catus* | Portugal | LC915109* |
| *Rickettsia asembonensis* | *Ctenocephalides felis* | *Canis lupus familiaris* | Portugal | LC915110* |
| *Rickettsia felis* | *Ctenocephalides felis* | *Felis catus* | Portugal | LC915111* |
| *Rickettsia felis* | *Ctenocephalides felis* | *Felis catus* | Portugal | LC915112* |
| *Rickettsia felis* | *Ctenocephalides felis* | *Felis catus* | Portugal | LC915113* |
| *Rickettsia felis* | *Ctenocephalides felis* | *Canis lupus familiaris* | Portugal | LC915114* |
| *Rickettsia felis* | *Ctenocephalides felis* | *Canis lupus familiaris* | Portugal | LC915115* |
| *Rickettsia felis* | *Ctenocephalides felis* | *Felis catus* | Portugal | LC915116* |
| *Rickettsia felis* | *Ctenocephalides felis* | *Canis lupus familiaris* | Portugal | LC915117* |
| *Rickettsia felis* | *Ctenocephalides felis* | *Felis catus* | Portugal | LC915118* |
| *Rickettsia felis* | *Ctenocephalides felis* | *Canis lupus familiaris* | Portugal | LC915119* |
| *Rickettsia felis* | *Ctenocephalides felis* | *Canis lupus familiaris* | Portugal | LC915120* |
| *Rickettsia felis* | *Ctenocephalides felis* | *Felis catus* | Portugal | LC915121* |
| *Rickettsia felis* | *Ctenocephalides felis* | *Canis lupus familiaris* | Portugal | LC915122* |
| *Rickettsia felis* | *Ctenocephalides felis* | *Felis catus* | Portugal | LC915123* |
| *Rickettsia felis* | *Ctenocephalides felis* | *Felis catus* | Portugal | LC915124* |
| *Rickettsia felis* | *Ctenocephalides felis* | *Canis lupus familiaris* | Portugal | LC915125* |
| *Rickettsia felis* | *Ctenocephalides felis* | *Canis lupus familiaris* | Portugal | LC915126* |
| *Rickettsia felis* | *Ctenocephalides felis* | *Canis lupus familiaris* | Portugal | LC915127* |
| *Rickettsia felis* | *Ctenocephalides felis* | *Felis catus* | Portugal | LC915128* |
| *Rickettsia felis* | *Ctenocephalides felis* | *Canis lupus familiaris* | Portugal | LC915129* |
| *Rickettsia felis* | *Ctenocephalides felis* | *Canis lupus familiaris* | Portugal | LC915130* |
| *Rickettsia felis* | *Ctenocephalides felis* | *Canis lupus familiaris* | Portugal | LC915131* |
| *Rickettsia felis* | *Ctenocephalides felis* | *Canis lupus familiaris* | Portugal | LC915132* |
| *Rickettsia felis* | *Ctenocephalides felis* | *Felis catus* | Portugal | LC915133* |
| *Rickettsia felis* | *Ctenocephalides felis* | *Canis lupus familiaris* | Portugal | LC915134* |
| *Rickettsia felis* | *Ctenocephalides felis* | *Canis lupus familiaris* | Portugal | LC915135* |
| *Rickettsia felis* | *Ctenocephalides felis* | *Canis lupus familiaris* | Portugal | LC915136* |
| *Rickettsia felis* | *Ctenocephalides felis* | *Canis lupus familiaris* | Portugal | LC915137* |
| *Rickettsia felis* | *Ctenocephalides felis* | *Felis catus* | Portugal | LC915138* |
| *Rickettsia felis* | *Ctenocephalides felis* | *Canis lupus familiaris* | Portugal | LC915139* |
| *Rickettsia felis* | *Ctenocephalides felis* | *Felis catus* | Portugal | LC915140* |
| *Rickettsia felis* | *Ctenocephalides felis* | *Canis lupus familiaris* | Portugal | LC915141* |
| *Rickettsia felis* | *Ctenocephalides felis* | *Canis lupus familiaris* | Portugal | LC915142* |
| *Rickettsia felis* | *Ctenocephalides felis* | *Felis catus* | Portugal | LC915143* |
| *Rickettsia felis* | *Ctenocephalides felis* | *Felis catus* | Portugal | LC915144* |
| *Rickettsia felis* | *Ctenocephalides felis* | *Canis lupus familiaris* | Portugal | LC915145* |
| *Rickettsia felis* | *Ctenocephalides felis* | *Felis catus* | Portugal | LC915146* |
| *Rickettsia felis* | *Ctenocephalides felis* | *Canis lupus familiaris* | Portugal | LC915147* |
| *Rickettsia felis* | *Ctenocephalides felis* | *Canis lupus familiaris* | Portugal | LC915148* |
| *Rickettsia felis* | *Ctenocephalides felis* | *Felis catus* | Portugal | LC915149* |
| *Rickettsia felis* | *Ctenocephalides felis* | *Canis lupus familiaris* | Portugal | LC915150* |
| *Rickettsia felis* | *Ctenocephalides felis* | *Canis lupus familiaris* | Portugal | LC915151* |
| *Rickettsia felis* | *Ctenocephalides felis* | *Canis lupus familiaris* | Portugal | LC915152* |
| *Rickettsia felis* | *Ctenocephalides felis* | *Canis lupus familiaris* | Portugal | LC915153* |
| *Rickettsia felis* | *Ctenocephalides felis* | *Canis lupus familiaris* | Portugal | LC915154* |
| *Rickettsia felis* | *Ctenocephalides felis* | *Felis catus* | Portugal | LC915155* |
| *Rickettsia felis* | *Ctenocephalides felis* | *Felis catus* | Portugal | LC915156* |
| *Rickettsia felis* | *Ctenocephalides felis* | *Canis lupus familiaris* | Portugal | LC915157* |
| *Rickettsia felis* | *Ctenocephalides felis* | *Felis catus* | Portugal | LC915158* |
| *Rickettsia felis* | *Ctenocephalides felis* | *Canis lupus familiaris* | Portugal | LC915159* |
| *Rickettsia felis* | *Ctenocephalides felis* | *Canis lupus familiaris* | Portugal | LC915160* |
| *Rickettsia felis* | *Ctenocephalides felis* | *Canis lupus familiaris* | Portugal | LC915161* |
| *Rickettsia felis* | *Ctenocephalides felis* | *Felis catus* | Portugal | LC915162* |
| *Rickettsia felis* | *Ctenocephalides felis* | *Canis lupus familiaris* | Portugal | LC915163* |
| *Rickettsia felis* | *Ctenocephalides felis* | *Canis lupus familiaris* | Portugal | LC915164* |
| *Rickettsia felis* | *Ctenocephalides felis* | *Canis lupus familiaris* | Portugal | LC915165* |
| *Rickettsia felis* | *Ctenocephalides felis* | *Canis lupus familiaris* | Portugal | LC915166* |
| *Rickettsia felis* | *Ctenocephalides felis* | *Felis catus* | Portugal | LC915167* |
| *Rickettsia felis* | *Ctenocephalides felis* | *Felis catus* | Portugal | LC915168* |
| *Rickettsia felis* | *Ctenocephalides felis* | *Canis lupus familiaris* | Portugal | LC915169* |
| *Rickettsia felis* | *Ctenocephalides felis* | *Felis catus* | Portugal | LC915170* |
| *Rickettsia felis* | *Ctenocephalides felis* | *Felis catus* | Portugal | LC915171* |
| *Rickettsia felis* | *Ctenocephalides felis* | *Canis lupus familiaris* | Portugal | LC915172* |
| *Rickettsia felis* | *Ctenocephalides felis* | *Canis lupus familiaris* | Portugal | LC915173* |
| *Rickettsia felis* | *Ctenocephalides felis* | *Felis catus* | Portugal | LC915174* |
| *Rickettsia felis* | *Ctenocephalides felis* | *Felis catus* | Portugal | LC915175* |
| *Rickettsia felis* | *Ctenocephalides felis* | *Canis lupus familiaris* | Portugal | LC915176* |
| *Rickettsia felis* | *Ctenocephalides felis* | *Felis catus* | Portugal | LC915177* |
| *Rickettsia felis* | *Ctenocephalides felis* | *Canis lupus familiaris* | Portugal | LC915178* |
| *Rickettsia felis* | *Ctenocephalides felis* | *Canis lupus familiaris* | Portugal | LC915179* |
| *Rickettsia felis* | *Ctenocephalides felis* | *Canis lupus familiaris* | Portugal | LC915180* |
| *Rickettsia felis* | *Ctenocephalides felis* | *Canis lupus familiaris* | Portugal | LC915181* |
| *Rickettsia felis* | *Ctenocephalides felis* | *Canis lupus familiaris* | Portugal | LC915182* |
| *Rickettsia felis* | *Ctenocephalides felis* | *Canis lupus familiaris* | Portugal | LC915183* |
| *Rickettsia felis* | *Ctenocephalides felis* | *Canis lupus familiaris* | Portugal | LC915184* |
| *Rickettsia felis* | *Liposcelis bostrychophila* | NA | USA | GQ385243 |
| *Rickettsia asembonensis* | *Ctenocephalides felis* | NA | Peru | MK923721 |
| *Rickettsia asembonensis* | *Ctenocephalides felis* | NA | Peru | MK923726 |
| *Rickettsia asembonensis* | *Ctenocephalides canis* | NA | Peru | MK923741 |
| *Rickettsia asembonensis* | *Ctenocephalides canis* | NA | Peru | MK923736 |
| *Candidatus* Rickettsia senegalensis | *Ctenocephalides felis* | NA | USA | KT304219 |
| *Candidatus* Rickettsia senegalensis | *Ctenocephalides felis* | NA | Senegal | KF666470 |
| *Candidatus* Rickettsia senegalensis | *Ctenocephalides felis* | NA | Colombia | MK548198 |
| *Rickettsia conorii* subsp. *israelensis* | NA | NA | NA | AF123712 |
| *Rickettsia conorii* subsp. *Caspia* | NA | NA | NA | AF123708 |
| *Rickettsia conorii* strain *India* | NA | NA | NA | AF123726 |
| *Rickettsia conorii* strain *Seven* | NA | NA | NA | AF123721 |
| *Rickettsia sibirica* | NA | NA | NA | AF123722 |
| *Rickettsia africae* | NA | *Homo sapiens* | Austria (imported from Tanzania) | KU721071 |
| *Rickettsia slovaca* | NA | NA | NA | AF123723 |
| *Rickettsia honei* | NA | NA | NA | AF123711 |
| *Candidatus* Rickettsia goldwasserii | *Haemaphysalis adleri* | *Canis aureus* | Israel | HM136926 |
| *Rickettsia japonica* | NA | NA | NA | AF123713 |
| *Rickettsia conorii* subsp. *heilongjiangensis* | NA | NA | NA | AY260451 |
| *Rickettsia massiliae* | NA | NA | NA | AF123714 |
| *Rickettsia conorii* subsp. *Raoultii* | NA | NA | NA | EU036984 |
| *Rickettsia aeschlimannii* | NA | NA | NA | AF123705 |
| *Rickettsia montanensis* | NA | NA | NA | AF123716 |
| *Rickettsia akari* | NA | NA | NA | AF123707 |
| *Rickettsia australis* | NA | NA | NA | AF123709 |
| *Rickettsia typhi* | NA | NA | NA | L04661 |
| *Rickettsia prowazekii* | NA | NA | NA | AF123718 |
| *Rickettsia helvetica* | NA | NA | NA | AF123725 |

***** Sequences generated in the present study; all other sequences were retrieved from GenBank.

NA, not available.

**Table S3**. Metadata of Dipylidium caninum 28S rDNA gene sequences included in the phylogenetic analysis.

| Species | Invertebrate host | Vertebrate host | Country | Accession number |
| --- | --- | --- | --- | --- |
| *Dipylidium caninum* | *Ctenocephalides felis* | *Canis lupus familiaris* | Portugal | LC914712* |
| *Dipylidium caninum* | *Ctenocephalides felis* | *Canis lupus familiaris* | Portugal | LC914713* |
| *Dipylidium caninum* | *Ctenocephalides felis* | *Canis lupus familiaris* | Portugal | LC914714* |
| *Dipylidium caninum* | *Ctenocephalides felis* | *Canis lupus familiaris* | Portugal | LC914715* |
| *Dipylidium caninum* | *Ctenocephalides felis* | *Canis lupus familiaris* | Portugal | LC914716* |
| *Dipylidium caninum* | *Ctenocephalides felis* | *Felis catus* | Portugal | LC914717* |
| *Dipylidium caninum* | *Ctenocephalides felis* | *Canis lupus familiaris* | Portugal | LC914718* |
| *Dipylidium caninum* | *Ctenocephalides felis* | *Canis lupus familiaris* | Portugal | LC914719* |
| *Dipylidium caninum* | *Ctenocephalides felis* | *Canis lupus familiaris* | Portugal | LC914720* |
| *Dipylidium caninum* | *Ctenocephalides felis* | *Canis lupus familiaris* | Portugal | LC914721* |
| *Dipylidium caninum* | *Ctenocephalides felis* | *Felis catus* | Portugal | LC914722* |
| *Dipylidium caninum* | *Ctenocephalides felis* | *Felis catus* | Portugal | LC914723* |
| *Dipylidium caninum* | *Ctenocephalides felis* | *Felis catus* | Portugal | LC914724* |
| *Dipylidium caninum* | *Ctenocephalides felis* | *Canis lupus familiaris* | Portugal | LC914725* |
| *Dipylidium caninum* | *Ctenocephalides felis* | *Canis lupus familiaris* | Portugal | LC914726* |
| *Dipylidium caninum* | *Ctenocephalides felis* | *Felis catus* | Portugal | LC914727* |
| *Dipylidium caninum* | *Ctenocephalides felis* | *Felis catus* | Portugal | LC914728* |
| *Dipylidium caninum* | *Ctenocephalides felis* | *Felis catus* | Portugal | LC914729* |
| *Dipylidium caninum* | *Ctenocephalides felis* | *Felis catus* | Portugal | LC914730* |
| *Dipylidium caninum* | *Ctenocephalides felis* | *Felis catus* | Portugal | LC914731* |
| *Dipylidium caninum* | *Ctenocephalides felis* | *Felis catus* | Portugal | LC914732* |
| *Raillietina australis* | NA | *Dromaeus novaehollandiae* | Australia | AF286914.1 |
| *Dipylidium caninum* | NA | NA | NA | MG575743 |
| *Dipylidium caninum* | NA | NA | NA | MG575741 |
| *Dipylidium caninum* | NA | NA | NA | MH045476 |
| *Dipylidium caninum* | NA | NA | NA | MH045481 |
| *Dipylidium caninum* | NA | NA | NA | MH040831 |
| *Dipylidium caninum* | *Ctenocephalides felis* | NA | Malaysia | KY751956 |
| *Dipylidium caninum* | NA | NA | NA | MG575742 |
| *Dipylidium caninum* | NA | NA | NA | MG575740 |
| *Dipylidium caninum* | NA | *Canis lupus familiaris* | USA | MH182476 |
| *Dipylidium caninum* | NA | *Canis lupus familiaris* | USA | MH18247 |
| *Dipylidium caninum* | NA | *Canis lupus familiaris* | USA | MH182478 |
| *Dipylidium caninum* | NA | *Canis lupus familiaris* | NA | MH040832 |
| *Dipylidium caninum* | NA | NA | NA | MH040851 |
| *Dipylidium caninum* | NA | NA | NA | MH040849 |
| *Dipylidium caninum* | NA | NA | NA | MH045467 |
| *Dipylidium caninum* | NA | NA | NA | AF023120 |

***** Sequences generated in the present study; all other sequences were retrieved from GenBank.

NA, not available.
