# Supplementary material for: Molecular detection of zoonotic pathogens in fleas collected from dogs and cats in Portugal
Source: Parasit Vectors. 2026 May 30;19:311. doi: 10.1186/s13071-026-07456-4 (PMC13435523; doi:10.1186/s13071-026-07456-4)
Supplement: Supplementary file 2 [file 13071_2026_7456_MOESM2_ESM.docx]

**Table S1** Potential risk factors associated with *Bartonella* spp. infection in fleas from dogs and cats in mainland Portugal.

| Variable (Reference category) | *β* | *SE* | Wald *χ^2^* | *df* | *P*-value | aOR (95% CI) |
| --- | --- | --- | --- | --- | --- | --- |
| Host (Dog) |  |  |  |  |  |  |
| Cat | 1.94 | 0.30 | 42.09 | 1 | <0.0001 | 6.95 (3.87-12.49) |
| Lifestyle (Domestic) |  |  |  |  |  |  |
| Shelter/stray | 0.56 | 0.24 | 5.55 | 1 | 0.019 | 1.75 (1.10-2.78) |
| Region - NUTS II (AML) |  |  |  |  |  |  |
| Norte | -0.80 | 0.30 | 7.05 | 1 | 0.008 | 0.45 (0.25-0.81) |
| Centro | -1.49 | 0.43 | 11.98 | 1 | 0.001 | 0.23 (0.10-0.53) |
| Alentejo | -1.03 | 0.36 | 7.96 | 1 | 0.005 | 0.36 (0.18-0.73) |
| Algarve | -2.29 | 0.46 | 24.2 | 1 | <0.0001 | 0.10 (0.04-0.25) |
| Constant | -2.95 | 0.38 | 58.9 | 1 | <0.0001 |  |
| Homer and Lemeshow test: *χ2* = 20.25, *df* = 8, *P* = 0.009; Likelihood ratio test (*G²*) = 112.75, *df* = 6, *P* <0.0001; Area under the curve = 0.79, *P* < 0.0001. | | | | | | |
| Abbreviations: OR, adjusted odds ratio; AML, Área Metropolitana de Lisboa; CI, Confidence interval; NUTS, Nomenclature of units for territorial statistics. **Table S2** Potential risk factors associated with *Rickettsia* spp. infection in fleas from dogs and cats in mainland Portugal. Variable (Reference category) \| *β* \| *SE* \| Wald *χ^2^* \| *df* \| *P*-value \| aOR (95% CI); Host (Dog) \| \| \| \| \| \|; Cat \| -0.36 \| 0.16 \| 5.27 \| 1 \| 0.022 \| 0.70 (0.51-0.95); Flea species (Others) \| \| \| \| \| \|; *C. felis* \| 1.28 \| 0.53 \| 5.88 \| 1 \| 0.015 \| 3.59 (1.28-10.09); Region - NUTS II (AML) \| \| \| \| \| \|; Norte \| -0.83 \| 0.27 \| 9.15 \| 1 \| 0.002 \| 0.44 (0.26-0.75); Centro \| -0.53 \| 0.29 \| 3.34 \| 1 \| 0.067 \| 0.59 (0.33-1.04); Alentejo \| -0.56 \| 0.31 \| 3.31 \| 1 \| 0.069 \| 0.57 (0.32-1.04); Algarve \| -0.67 \| 0.29 \| 5.17 \| 1 \| 0.023 \| 0.51 (0.29-0.91); Constant \| -22.09 \| 0.58 \| 12.16 \| 1 \| <0.0001 \|; Homer and Lemeshow test: *χ2* = 10.06, *df* = 8, *P* = 0.261; Likelihood ratio test (*G²*) = 21.71, *df* = 6, *P* = 0.001; Area under the curve = 0.60, *P* < 0.0001. \| \| \| \| \| \|; *Statistically significant at *α* = 0.05. Abbreviations: aOR, adjusted odds ratio; AML, Área Metropolitana de Lisboa; CI: Confidence interval, NUTS: Nomenclature of units for territorial statistics. \| \| \| \| \| \| | | | | | | |
